# Topography-based implants for bone regeneration: Design, biological mechanism, and therapeutics

**DOI:** 10.1016/j.mtbio.2025.102066

**Published:** 2025-07-13

**Authors:** Weiwei Guo, Zuge Yang, Fuwei Liu, Jianye Song, Wenhao Yang, Yunpeng Li, Wenhui Hu, Kun Wang

**Affiliations:** a Department of Stomatology, Xinqiao Hospital, Third Military Medical University, Chongqing, 400037, PR China; b State Key Laboratory of Oral & Maxillofacial Reconstruction and Regeneration, National Clinical Research Center for Oral Diseases, Shaanxi Clinical Research Center for Oral Diseases, Department of Oral and Maxillofacial Surgery, School of Stomatology, The Fourth Military Medical University, Xi'an, Shanxi, 710032, PR China; c Department of Stomatology, Southern Theater Air Force Hospital, Guangzhou, Guangdong, 510000, PR China; d Department of Basic Medicine, Frontier Medical Service Training Brigade, Third Military Medical University, Changji, Xinjiang, 831200, PR China; e Department of Biomedical Materials Science, Third Military Medical University, Chongqing, 400038, PR China

**Keywords:** Implants, Bone regeneration, Surface morphology, Biological mechanisms, Translational applications

## Abstract

With the increasing demand for bone defect repair, bone implant materials have emerged as a critical alternative to traditional autologous or allogeneic bone grafts. However, their clinical performance remains limited due to challenges such as prolonged healing times and suboptimal repair quality. Moreover, in patients with certain pathological conditions (e.g., diabetes mellitus and osteoporosis), disruptions in the bone microenvironment further compromise regenerative outcomes. To address these limitations, surface modification strategies have been developed to regulate implant-bone tissue interactions and improve therapeutic efficacy. This review systematically summarizes recent advances in bone regeneration implants with a focus on topographical modifications, encompassing design principles, underlying biological mechanisms, and therapeutic applications. Particular attention is given to the influence of implant surface topography on the biological behaviors of osteoblasts, osteoclasts, and macrophages within the bone microenvironment, as well as their responses under complex pathological and physiological conditions. The review also discusses current challenges related to achieving micro/nanoscale structural balance, personalization, and clinical translation of implant surface topographies, and highlights future directions in precision bone regeneration through multidisciplinary approaches, artificial intelligence-driven optimization, and long-term clinical validation. Collectively, these insights may inform future research on bone implant materials and support the development of novel strategies for personalized treatment of bone defect repair.

## Highlights

• In-depth investigation of implant surface topography effects on biological functions of various cells in bone microenvironment.
• Topography-modified implants enhance bone regeneration in complex conditions by modulating cell activity.
• Challenges and future prospects of topography-based implants for bone regeneration.

## Graphical abstract

**Figure:**
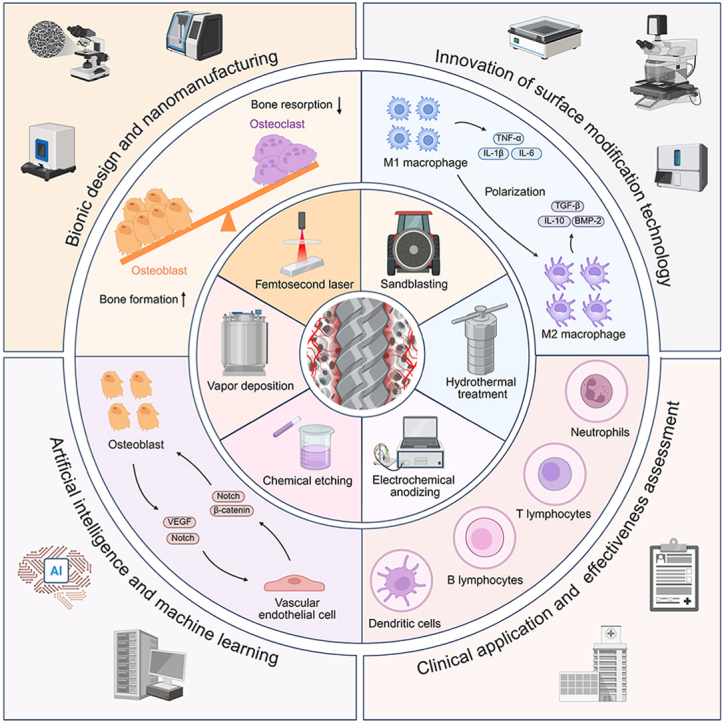

## 1 Introduction

The number of bone grafting procedures continues to increase annually due increase in the aging population and the incidence of conditions such as trauma, inflammation, tumor resection, and congenital malformations [[1], [2], [3]]. Autologous bone grafting remains the gold standard for bone defect repair; however, it is associated with notable limitations, including high donor site morbidity, prolonged recovery time, and insufficient graft volume [[4], [5], [6]]. As a result, bone implant materials have emerged as promising alternatives and are now widely applied in orthopedics and dentistry [[7], [8], [9]]. It is estimated that nearly 300,000 patients in the United States undergo dental implantation annually, and over 200,000 hip replacement surgeries are performed each year [10]. Nevertheless, current bone implant materials still encounter significant challenges, including insufficient biocompatibility, limited bone regeneration capacity, and poor long-term stability, particularly under complex pathological conditions [[11], [12], [13], [14], [15]]. Additionally, reducing the inflammatory response and risk of infection while promoting rapid and stable bone regeneration remains an urgent clinical need [16,17].

The process from implantation to bone remodeling represents a temporally regulated and biologically complex sequence of events involving multiple cell types, such as bone marrow mesenchymal stem cells (BMSCs), osteoblasts, osteoclasts, and immune cells, including macrophages, all of which play essential roles in osteogenesis [18,19]. Upon implantation, a hematoma typically forms at the bone-implant interface [20], which rapidly recruits and activates immune cells, including neutrophils and macrophages, thereby initiating the early inflammatory response [21]. These immune cells are responsible for clearing necrotic tissue and debris and secreting various growth factors and cytokines that contribute to the transition from a pro-inflammatory to an anti-inflammatory microenvironment [22]. Subsequently, BMSCs and other progenitor cells differentiate into osteoblasts around the implant surface and initiate new bone formation [23]. Ultimately, osteoclasts resorb the redundant bone matrix, and osteoblasts deposit mature lamellar bone, leading to complete bone remodeling [24,25]. Throughout this process, the surface properties of the bone implant play an essential role in regulating host cell behaviors, such as adhesion, differentiation, and apoptosis, within the bone microenvironment, thereby directly influencing the efficacy of bone repair [26].

Surface modification strategies for bone implants primarily fall into two categories: bio-coatings and morphological modifications [[27], [28], [29], [30]]. Although bio-coatings, including hydroxyapatite, can enhance osteogenic outcomes via biochemical cues, their application is limited by inherent shortcomings [[31], [32], [33], [34], [35], [36]]. Conventional coating techniques such as plasma spraying often lead to undesirable features like grain coarsening, microcracks, and porosity, which weaken the bond strength and increase the risk of implant detachment, thereby hindering bone repair [[37], [38], [39]]. Moreover, although incorporating bioactive factors into coatings can improve implant bioactivity, such modifications raise concerns regarding toxicity and biological contamination, necessitating extensive preclinical validation for safety and efficacy [40,41]. In contrast, morphological modifications avoid the limitations associated with surface coatings and instead enhance osteogenesis by precisely regulating implant surface topography. These modifications stimulate key signaling pathways at the implant-bone interface, thereby significantly improving bone regeneration [[42], [43], [44], [45], [46]]. Such strategies address the limitations of traditional implants in terms of biosafety and lack of multifunctionality [[47], [48], [49], [50], [51]]. Furthermore, implant materials that mimic the composition and microarchitecture of native bone are increasingly recognized as optimal candidates for bone defect repair [52]. With the advancement of precision manufacturing technologies and bioinspired design principles, morphology-modified implants are poised to become a central solution for repairing complex bone defects [53,54].

Topography-engineered implants have been developed to address bone defects under diverse pathophysiological conditions and varying implantation time points [55,56]. The core design principle is to modulate the activity of osteoblasts and immune cells within the bone microenvironment through distinct surface structures, thereby promoting bone regeneration [[57], [58], [59], [60], [61]]. For instance, Wang et al. [62] demonstrated that hierarchical microgroove/nanotube structures promoted osteoblast differentiation via activation of the Wnt/β-catenin signaling pathway. Similarly, He et al. [63] reported that titanium nanotubes with a diameter of approximately 30 nm inhibited osteoclast differentiation through the integrin β1/FAKpY397/MAPK signaling axis, thereby enhancing early osteogenesis. Xu et al. [64] found that titanium nanotubes of approximately 140 nm in diameter could promote M2 polarization of macrophages and increase VEGF secretion through activation of the ERK1/2 and PI3K/Akt pathways. Moreover, surface-modified implants have shown favorable biocompatibility and osseointegration under pathological conditions such as diabetes and osteoporosis [[65], [66], [67]].

Despite significant advances, there is a lack of systematic reviews addressing surface topography design, associated biological mechanisms, and translational applications of bone implants [28,68,69]. Therefore, this review aims to comprehensively summarize current progress in the field, including implant-bone microenvironment interactions, topography fabrication techniques, the interplay between surface morphology and biological signaling pathways, in vivo therapeutic applications, and outstanding challenges. By doing so, we seek to provide a theoretical framework and practical guidance for the personalized design of bone implants and their application under complex pathological conditions.

## 2 Implant-bone microenvironment interactions regulate bone repair processes

**Fig. 1:**
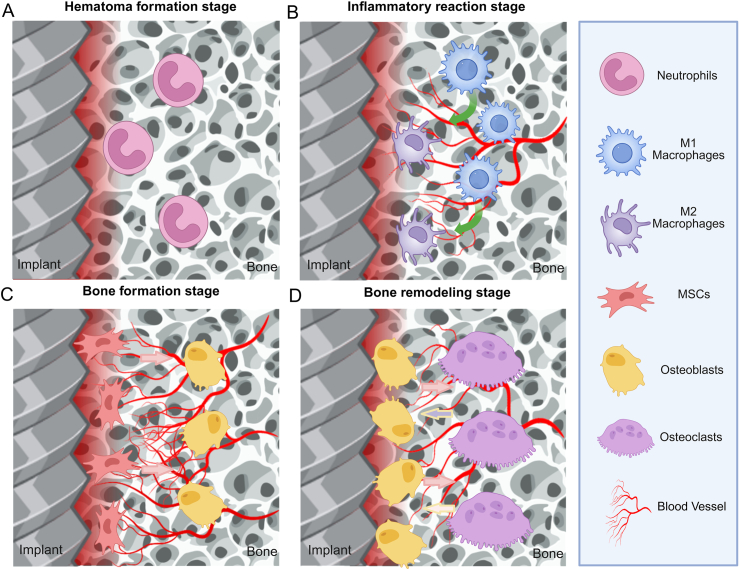
Schematic illustration of implant-bone microenvironment interactions regulating bone repair processes. A) Hematoma formation stage. B) Inflammatory reaction stage. C) Bone formation stage. D) Bone remodeling stage. Created in BioRender. Abbreviations: MSCs: Mesenchymal stem cells.

The process from the insertion of a bone implant into the body to the completion of bone remodeling involves a sequence of damage and subsequent repair, characterized by complexity and the involvement of various cell types. Among these, BMSCs contribute directly to osteogenesis, while osteoblasts and osteoclasts participate in bone formation and resorption, respectively. In addition, numerous immune cells, particularly macrophages, play essential roles in mediating the repair response [70]. The interaction between bone implants and the surrounding microenvironment is largely governed by the specific macroscopic and microscopic features of the implant surface, which in turn influence the bone repair process. Generally, the sequence of events in implant-mediated bone repair consists of four overlapping and consecutive phases, that is, hematoma formation, the inflammatory phase, scab formation, and tissue remodeling [23,71] (Fig. 1A and D).

### 2.1 Hematoma formation stage

When a bone implant enters the body, it can cause bone damage and rupture of blood vessels to a certain extent, leading to the rapid accumulation of blood at and around the injury site and the subsequent formation of a hematoma [20]. Hematoma formation plays a key role in bone repair by providing a stable structural framework that supports new bone formation. In addition, the associated histochemical processes promote osteoblast migration and the development of bridging bone callus, and the hematoma facilitates the recruitment of cells to the implant surface to establish the physiological basis necessary for effective bone regeneration [72,73]. Moreover, hematomas can rapidly recruit and activate various innate immune cells, such as neutrophils, lymphocytes, and macrophages. In response to fibrin deposition and immune cell activity, activated platelets release a cascade of chemokines and cytokines that initiate the early inflammatory response [21].

Implant surface characteristics play a pivotal role in hematoma formation and the subsequent progression, primarily by influencing the rate of hematoma development, its internal composition, and, consequently, the associated inflammatory response, cellular behavior, and bone repair processes [74,75]. To enhance these outcomes, implant surfaces are often specially treated to accelerate hematoma formation, modulate its organization and promote the release of growth factors. In this regard, Milleret et al. fabricated super hydrophilic sandblasted and acid-etched titanium surfaces by alkali treatment to facilitate the orderly arrangement of blood components in hematomas, which was reported to not only create a favorable microenvironment for cell migration but also served as a reservoir for signaling molecules and nutrients, thereby promoting the migration and differentiation of BMSCs [76]. Furthermore, nano-scale features on implant surfaces, such as titanium nanotubes, have been shown to significantly influence the inflammatory response and cellular morphology mediated by hematomas. Modifications to the nanotube structure can regulate these responses to create an environment favorable for bone immunomodulation and enhance the osteogenic process [77]. KieuNguyen et al. demonstrated that nano-topographical features on implant surfaces can significantly alter the expression profiles of long non-coding RNAs (lncRNAs) in hematomas to suppress osteoblastic differentiation and modulate key inflammatory mediators, including tumor necrosis factor (TNF) and nuclear factor-κB (NF-κB). Through these signaling pathways, the expression of mRNAs associated with bone resorption and inflammation could be downregulated, which ultimately influenced osseointegration through multiple molecular targets [78].

### 2.2 Inflammatory reaction stage

After implant placement, the inflammatory response plays a vital role in initiating bone formation and promoting osseointegration. In the early phase, immune cells become activated, and neutrophils rapidly migrate to the implant surface. These neutrophils secrete various cytokines, such as interleukin 1β (IL-1β), CXCL1-3, and TNF-α, which facilitate the recruitment of additional immune cells, including macrophages. Moreover, neutrophils release reactive oxygen species, which contribute to the regulation of the immune response [79,80]. In the early stages of inflammation, neutrophils are the first immune cells to infiltrate the healing tissue and initiate the immune response, followed sequentially by macrophages, lymphocytes, and other immune cells in a coordinated manner [21]. These infiltrating cells enter the hematoma and secrete growth factors and cytokines that facilitate the recruitment of mesenchymal cells. Moreover, neutrophils and macrophages clear the dead cells and debris and also release factors that promote the recruitment of mesenchymal progenitor cells from the periosteum, bone marrow, and systemic circulation [81]. A timely transition of the inflammatory microenvironment from a pro-inflammatory to an anti-inflammatory state is favorable for osteogenesis [22]. Macrophages play a key role in bone repair and can polarize into either the M1 or M2 phenotype in response to stimulation by the physicochemical properties of the implant surface [82,83]. Generally, M1 macrophages promote inflammation, contribute to tissue damage, and impede wound healing, whereas the M2 phenotype exhibits anti-inflammatory, pro-angiogenic, and pro-repair functions. M2 macrophages secrete cytokines such as transforming growth factor-β (TGF-β) and bone morphogenetic protein-2 (BMP-2), which support osteogenesis [84,85]. However, M1 macrophages are also essential during the early inflammatory phase, where they contribute to debris clearance, the establishment of the inflammatory microenvironment, and the initiation of angiogenesis [86,87]. A moderate inflammatory response during the early stages of bone repair has been shown to aid in infection control and promote new bone formation [88,89]. In the later stages of inflammation, M2-type macrophages become the predominant population both in number and duration, contributing to the resolution of inflammation and actively promoting osteoinduction and osteogenesis [90,91]. In parallel, other immune cells, including dendritic cells (DCs), T cells, and B cells, also participate in the inflammatory response and interact with one another, collectively influencing the process of osseointegration [[92], [93], [94]].

Various surface properties of the implant, including morphology [95,96], chemical composition [97,98], porosity [99], and stiffness [[100], [101], [102]], can influence the inflammatory response, and among these, the direction of macrophage polarization plays a particularly important role in determining the outcome of bone repair [103]. Both nanoscale and micro-nano-structured titanium surfaces have been shown to reduce macrophage adhesion and decrease the secretion of pro-inflammatory cytokines, such as IL-1β, IL-6, and TNF-α, in comparison with flat surfaces [104]. Luu et al. reported that micro- and nano-patterned grooves influence macrophage elongation, with the most pronounced effect observed at groove widths of 400–500 nm. Although surface notches do not significantly impact inflammatory activation, they facilitate the development of an anti-inflammatory, pro-healing macrophage phenotype [105]. Similarly, Wang et al. demonstrated that titanium surfaces with nanotube diameters of 30 nm are more effective in promoting M2-type polarization in mouse bone marrow-derived macrophages (BMMs) than surfaces with 100 nm diameter nanotubes [106].

### 2.3 Bone formation stage

This phase represents an important period in bone repair, during which both bone progenitor cells and BMSCs differentiate directly into osteoblasts around the bone implant and subsequently initiate intramembranous ossification [23]. Osteoblast adhesion marks the initial step in bone formation, followed by gradual proliferation, differentiation, maturation, and the formation of relatively loose woven bone. Throughout this process, the biocompatibility of the implant plays a crucial role, as the surface materials must be capable of eliciting appropriate bone tissue responses. The adhesion of osteogenic precursor cells and pre-osteoblasts to the implant is regulated by adhesion molecules such as integrins, which then trigger new bone formation [[107], [108], [109], [110]]. Bone formation is also closely linked to both the inflammatory response and angiogenesis, with neovascularization providing newly formed bone tissue with essential nutrients and oxygen, while also serving as a primary route for cellular migration [20,[111], [112], [113], [114]]. As osteoblasts continue to mature, they become progressively embedded in a mineralized extracellular matrix (ECM) and eventually calcify into bone [115]. During this phase, osteoblasts and osteoclasts function cooperatively to remodel the initially formed loose woven bone into structurally mature lamellar bone [23,116,117].

The surface characteristics of the implant exert a significant influence during the formation of the bone scab. It is widely recognized that rough surfaces facilitate osteoblast differentiation and proliferation while also suppressing osteoclast activity to enhance bone regeneration [118]. It has been shown that micro-scale structures provide appropriate roughness and bone-locking capacity, whereas nanoscale structures offer superior promotion of cell proliferation and differentiation [119]. Dalby et al. reported that mildly disordered nanopit structures were able to induce osteogenic differentiation of mesenchymal stem cells (MSCs) even in the absence of exogenous inducing factors [120]. Rivera et al. demonstrated that nanotube structures enhance the adsorption of fibronectin and biliprotein, thereby increasing the number of osteoblasts adhering to the surface [121]. Similarly, Cunha et al. found that laser-generated nanotextured surfaces promoted matrix mineralization and the formation of bone-like nodules more effectively than polished or micropillar-textured surfaces [122]. Xu et al. further showed that micro/nano-mesh structures, which integrate the advantages of both micro- and nano-scale morphologies, enhanced the elongation of cellular filamentous pseudopodia, osteoclast differentiation, and longer cytoskeleton [123].

### 2.4 Bone remodeling stage

Bone repair progresses into the remodeling phase with the gradual replacement of woven bone by lamellar bone and the initiation of resorption of excess bone crust. During this stage, the activity of osteoclasts surpasses that of osteoblasts, leading to the resorption or replacement of nonessential bone matrix components [24]. Osteoclasts resorb the initially formed woven bone, while osteoblasts synthesize mature lamellar bone to optimize the structural and mechanical properties of bone tissue, which facilitates bone tissue renewal, remodeling, and the maintenance of structural integrity of bones [23,25,116,117]. The entire remodeling process is closely regulated by continuous interactions between bone tissue and the implant surface [26]. Numerous signaling molecules are involved in orchestrating the functional balance between osteoblasts and osteoclasts, among which the receptor activator of nuclear factor-κB ligand (RANKL) and osteoprotegerin (OPG) have been found to play essential roles in mediating cellular communication to maintain a dynamic equilibrium between bone resorption and bone formation, which is essential for successful bone remodeling [25,117,124].

The surface morphology of bone implants significantly affects the remodeling phase by modulating cellular behavior, influencing associated signaling pathways, and altering the immune microenvironment. Silverwood et al. demonstrated that titanium dioxide nanopillar structures can inhibit osteoclast activity and differentiation [125]. Conversely, it has also been reported that titanium surfaces with moderate roughness (e.g., Ra ∼1.25 μm) can promote osteoclast differentiation [126]. Moreover, the microtopography of titanium surfaces can influence the organization of osteoclast resorption structures [127,128]. Surface topography has also been shown to regulate the activity of inflammasomes, the NF-κB signaling pathway, and Toll-like receptors (TLRs), all of which are involved in osteoclastogenesis and bone remodeling [[129], [130], [131], [132]]. For example, rough hydrophilic titanium surfaces have been shown to reduce the activation of the NF-κB signaling pathway, decrease osteoclast numbers, and increase bone-implant contact (BIC) [131]. In systemic disease conditions such as osteoporosis and diabetes mellitus, implant surface morphologies with immunomodulatory properties can enhance osseointegration by regulating protein-implant interactions, alleviating inflammation, promoting bone formation, and suppressing osteoclastogenesis. For instance, nanostructured titanium surfaces have been shown to improve osseointegration in experimental models of diabetes and osteoporosis. Similarly, hydrophilic microrough surfaces have demonstrated the ability to enhance immune function and facilitate bone healing in a type 2 diabetes model [65,[133], [134], [135]].

## 3 Preparation of bone implant surface topography

The surface topography of bone implants encompasses structural features such as roughness, shape, texture, and porosity. These characteristics influence a range of biological processes, including cell adhesion, proliferation, differentiation, apoptosis, and protein adsorption at the implant interface, all of which collectively affect the osteogenic performance of the implant [136]. Current surface topography modification strategies are broadly categorized into physical and chemical approaches. Physical modification methods include sandblasting, laser treatment, and additive manufacturing, while chemical modification techniques involve processes such as chemical etching, micro-arc oxidation, anodization, and hydrothermal treatment (Table 1).

**Table 1:** Preparation methods of bone implant surface topography.

Topography preparation methods	Technology used	Topographical features	Factors affecting morphology	Advantages of preparation technology	Disadvantages of preparation techniques	Ref.
Physical preparation methods	Sandblasting	Formation of tiny pits and cracks, increased surface roughness	Size and shape of sandblasted particles, sandblast pressure, sandblast time, sandblast angle	Improved surface roughness and biological activity	Contamination caused by abrasive particle residue, surface damage due to excessive blasting	[[137], [138], [139], [140], [141], [142], [143], [144]]
	Laser	Formation of micro- or nano-scale fine structures such as grooves, pits, micropores	Laser pulse energy, pulse width, scanning speed, repetition frequency, focused spot size	High precision, processing of complex structures with little material damage, suitable for a wide range of materials, pollution-free	Slower processing speed, low efficiency, high equipment cost	[[145], [146], [147], [148], [149], [150], [151], [152]]
	Selective laser melting	Formation of porous or dense structures with adjustable surface roughness for precise control of microstructure	Laser power, scanning speed, powder layer thickness, exposure time, powder material	Personalized customization, suitable for various materials, high-precision manufacturing	High equipment costs, low production efficiency, powder material residue, lack of long-term mechanical research	[[153], [154], [155], [156], [157]]
	Electron beam melting	Formation of controlled dense and homogeneous microstructures	Electron beam current, voltage, scanning speed, melting time, powder particle size	High material purity, low residual stress, and minimal deformation	narrow range of materials, powder material residue, lack of long-term mechanical research	[157,158]
Chemical preparation methods	Chemical etching	Formation of rough surface features on the micron or nanometer scale, irregular porous structure	Etching temperature, etching rate, etchant concentration and chemical formulation	Relatively simple to operate, no need for complex equipment, low cost	Material loss is uncontrollable, excessive etching is likely to occur, acidic or alkaline liquids may remain, etching of complex implant surfaces is uneven	[[159], [160], [161], [162]]
	Micro-arc oxidation	Microporous structure with increased surface roughness	Electrolyte composition, voltage, current, processing time, temperature	Dense ceramic layer, high hardness, good wear and corrosion resistance, good biocompatibility	Parameters with difficult and precise control, the process is expensive and complicated	[[163], [164], [165]]
	Anodizing	Controllable pore arrangement and film thickness, pore diameter and arrangement density determined by oxidation current density and time	Sulfuric acid concentration, tank temperature, oxidation voltage, oxidation current density	Precisely regulated morphology, good reproducibility, simple and economical process	Mechanical stability may be reduced, and the bond strength between the oxide film and the substrate is relatively poor	[[166], [167], [168], [169]]
	Hydrothermal treatment	Regular microstructures such as nanosheets or rods	Reaction temperature, reaction time, reactant concentration and pressure	Simple and economical process, suitable for complex shapes of implants, with good uniformity	Long reaction times and relatively low productivity, need for precisely controlled reaction conditions	[[170], [171], [172], [173], [174]]

### 3.1 Physical surface modification

#### 3.1.1 Sandblasting

Sandblasting is a surface modification technique in which abrasive particles (e.g., Al_2_O_3_, TiO_2_, or SiO_2_) are projected onto the implant surface via a high-pressure air stream, generating macroscopic surface roughness [137,138]. By adjusting parameters such as particle type, size, jetting pressure, and duration, the surface morphology can be optimized to enhance osteoinductive potential and improve surface wettability, thereby promoting osteoblast adhesion [139,140]. Although sandblasting is one of the most established surface modification techniques [141,142], it presents certain limitations. The process requires precise control of multiple variables, and residual abrasive particles may result in microbial contamination, necessitating rigorous post-treatment cleaning [143]. Moreover, long-term observations have indicated that specific sandblasted surface patterns may increase the risk of peri-implantitis and implant failure [144].

#### 3.1.2 Laser

**Fig. 2:**
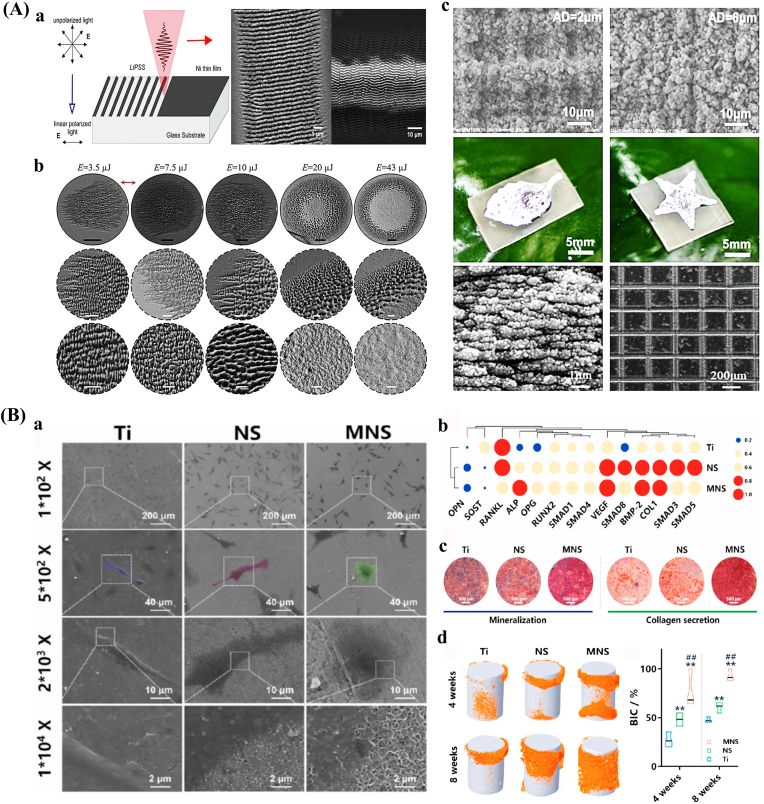
Surface morphology of bone implants prepared by physical and chemical methods. (A) Fabrication and applications of surface micro/nanostructures by femtosecond laser. a. Schematic diagram. b. SEM images with different pulse energies. c. SEM images after femtosecond laser treatment and photographs of liquid metal patterns of different shapes [148]. (B) Micro/nano-mimetic morphology preparation and promotion of bone integration in rabbits. a. Morphology of BMSCs on bionic surfaces. b. Osteogenic gene expression. c. Qualitative images of mineralization formation and collagen type I. d. 3D Micro-CT images and quantitative results of BIC [172]. Abbreviations: LIPSS: Laser-induced periodic surface structures; SEM: Scanning electron microscope; BMSCs: Mesenchymal stem cells; Micro-CT: Micro-computed tomography; BIC: Bone-to-implant contact.

Laser texturing enables precise control over the size, shape, and spatial distribution of surface features at both the micro- and nanoscale, resulting in highly uniform and reproducible surface modifications [145,146]. Femtosecond lasers represent a high-precision approach characterized by ultrashort pulse durations and tightly focused beam spots, with peak power densities surpassing the optical damage threshold of all known solid materials. This allows for selective removal or modification of micro-regions in a wide range of substrates and facilitates the fabrication of complex micro/nanostructures [[147], [148], [149]] (Fig. 2A). Multiphoton absorption is a key mechanism for the generation of surface micro/nanostructures by femtosecond lasers, which usually occurs in high-intensity laser fields [150]. Laser texturing eliminates the need for harsh chemicals or solvents, thus minimizing the risk of contamination and simplifying the processing workflow [151]. However, laser-based methods are time-intensive, and the fabrication of three-dimensional micro- and nanostructures remains technically challenging [152].

#### 3.1.3 Additive manufacturing

Additive manufacturing (AM), commonly referred to as 3D printing, is an advanced layer-by-layer fabrication technique that enables the rapid production of complex geometries guided by computer-aided design (CAD) and allows the construction of patient-specific three-dimensional metal scaffolds with precisely controllable architectures [153,154]. The primary AM techniques applied in bone implant fabrication include selective laser melting (SLM) and electron beam melting (EBM).

Selective laser melting (SLM) employs a high-energy focused laser beam to selectively irradiate pre-deposited metal powders, inducing localized melting and subsequent solidification. This process is conducted in an inert gas atmosphere, enabling the production of complex, customized implants with high dimensional accuracy [155,156]. SLM is compatible with a broad range of biocompatible materials, particularly titanium alloys. By manipulating parameters such as laser power and scanning speed, the microstructure and overall properties of the implant can be finely tuned to meet stringent clinical requirements. However, SLM technology may leave powder material residues, lacks detailed research on long-term performance based on fracture mechanics, and has issues with high equipment costs and low production efficiency in large-scale clinical applications, limiting its widespread use [155,157].

Electron beam melting (EBM) utilizes electron beams to melt metal powders within a vacuum environment, which facilitates precise control over interstitial element content and yields high material purity [158]. The uniform heating during EBM processing results in low residual stress and minimal component distortion. The disadvantage is that, due to the closed system enforced by the original equipment manufacturer, EBM research is limited to a narrow range of materials. There are also issues such as powder material residue and a lack of detailed research on long-term performance based on fracture mechanics [157].

### 3.2 Chemical surface modification

#### 3.2.1 Chemical etching

Chemical etching is a surface modification technique in which the surface of bone implants is selectively dissolved using acidic or alkaline solutions to generate specific topographies that effectively remove machining-related contaminants such as grease and oxidized layers, thereby improving biocompatibility. Acidic treatments (e.g., HF/HNO_3_) and alkaline treatments (e.g., NaOH) can induce microporous structures that facilitate osseointegration [159]. Chemical etching does not require complex equipment and is amenable to large-scale production. The morphology and dimensions of the resulting micro/nanostructures can be controlled by adjusting the composition, concentration, and temperature of the etching solution [160,161]. However, chemical etching presents several limitations. Material loss during etching is difficult to control, and excessive etching can lead to dimensional inaccuracies and compromised mechanical properties. Furthermore, residual acids or alkalis may adversely affect cellular activity, and achieving uniform etching on implants with complex geometries can be challenging [159,162].

#### 3.2.2 Micro-arc oxidation

Micro-arc oxidation is a technique that forms ceramic oxide coatings on metal surfaces via high-voltage electrical discharge. The properties of the resulting coatings, such as surface roughness, porosity, and morphology, can be modulated by altering the electrolyte composition, applied voltage, current density, and treatment duration [163]. Surfaces treated with micro-arc oxidation typically exhibit a nanostructured porous morphology. At higher duty cycles, the coating develops micron-scale grooves interconnected with uniformly distributed nanopores. These hierarchical structures have been shown to enhance cell proliferation [164,165]. Despite its bioactivity-enhancing effects, the clinical application of micro-arc oxidation remains limited due to its high cost and procedural complexity. Additionally, inconsistencies in coating uniformity and thickness—dependent on the substrate material and process parameters—may compromise the reliability and performance of the implants [163].

#### 3.2.3 Anodizing

Anodizing is an electrochemical process in which the bone implant acts as the anode and a lead or platinum plate serves as the cathode. Under an applied electric field, ions in the electrolyte migrate toward the anode, where redox reactions generate micro- and nanostructures on the implant surface [166,167]. Studies have demonstrated that nanotubes of appropriate diameter and spacing can enhance cellular adhesion, migration, and osteogenic differentiation. The geometry of these nanotubes, specifically diameter, depth, and spacing, is influenced by factors such as voltage, anodization time, and electrolyte composition. In general, higher voltages and longer durations result in larger and deeper nanotubes [168]. Due to its simplicity, cost-effectiveness, and versatility, anodizing is widely employed for surface modification of bone implants. However, although favorable biological outcomes have been reported, the mechanical stability of anodized implants may be reduced [169].

#### 3.2.4 Hydrothermal treatment

Hydrothermal treatment is a straightforward and economical surface modification method that utilizes distilled water, often in combination with concentrated sodium hydroxide solutions [170]. This process facilitates the formation of bioactive coatings with various micro- and nanostructures, including nanopins, nanorods, and fibrous morphologies [171]. Short treatment durations (e.g., 30 s) with low alkali concentrations (e.g., 0.05 M) can generate moderately rough surfaces, whereas extended treatment periods (at least 24 h) under high alkali concentrations (5–10 M) and elevated temperatures (40–70 °C) promote the formation of micro/nanoscale structures [172] (Fig. 2B). Hydrothermal treatment is also suitable for three-dimensional porous orthopedic implants, as it enables comprehensive surface modification throughout the implant's architecture [173]. Nonetheless, the method has inherent limitations, including prolonged reaction times, relatively low productivity, and the need for precise control of reaction parameters to ensure consistent surface morphology [174].

## 4 Mechanism of signaling pathways in the implant-bone microenvironment

### 4.1 Osteoblasts

**Fig. 3:**
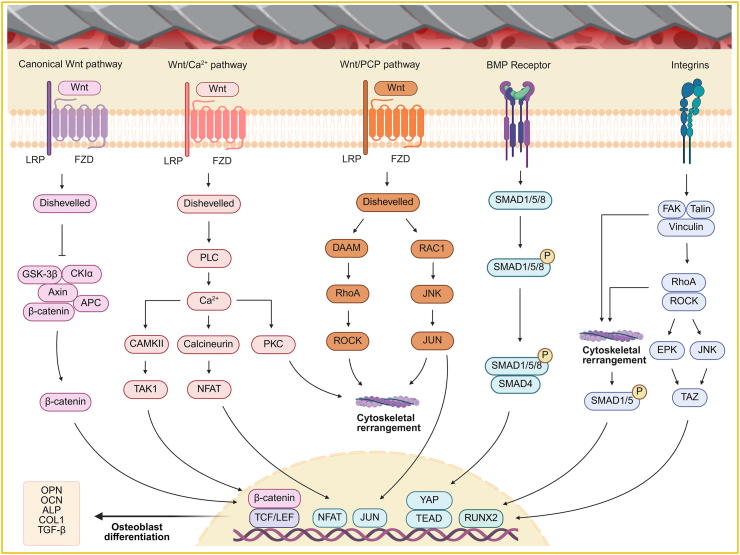
Mechanisms of osteoblast signaling pathways in the implant-bone microenvironment. Created in BioRender. Abbreviations: LRP: Low-density lipoprotein receptor-related protein; FZD: Frizzled; APC: Adenomatous polyposis coli; PLC: Phospholipase C; PKC: Protein kinase C; TAK1: TGF-β activated kinase 1; CAMKII: Calcium/calmodulin-dependent protein kinase II; NFAT: Nuclear factor of activated t-cells; DAAM: Dvl-associated activator of morphogenesis 1; RAC1: Ras-related C3 botulinum toxin substrate 1; JNK: c-Jun N-terminal kinase; ROCK: Rho-associated protein kinase; PCP: Planar cell polarity; BMP: Bone morphogenetic proteins; SMAD: Small mother against decapentaplegic; FAK: Focal adhesion kinase; TGF-β: Transforming growth factor-β; TCF: T-cell factor; LEF: Lymphoid enhancer-binding factor; RUNX2: Runt-related transcription factor 2; OPN: Osteopontin; OCN: Osteocalcin; ALP: Alkaline phosphatase; COL1: Collagen type 1.

The surface morphology of bone regeneration implants plays a key role in regulating the biological behavior of osteoblasts, including adhesion, differentiation, intercellular interaction, and apoptosis—processes that are essential for bone tissue regeneration and repair. Appropriate surface features enhance osteoblast adhesion to the implant and promote cellular morphological changes and maturation, thereby enhancing their differentiation and mineralization capacity. In parallel, optimized surface morphology strengthens interactions between osteoblasts, regulates signaling and metabolic activities, and promotes the maintenance of dynamic bone remodeling balance. In addition, surface properties influence osteoblast apoptosis, which further affects bone quality and the outcome of bone regeneration (Fig. 3).

#### 4.1.1 Osteoblast adhesion

The adhesion of osteoblasts is the primary step in osteogenesis after implantation of bone biomaterials and directly influences the quality and efficiency of early bone reconstruction [[175], [176], [177]]. The adhesion process of osteoblasts can be divided into two phases: the attachment phase and the adhesion phase [109]. The attachment phase occurs rapidly over a short period and primarily involves physical and chemical interactions between the cell and the material surface, such as ionic forces and van der Waals forces [178,179]. In contrast, the adhesion phase lasts longer and involves the participation of various biomolecules, including ECM proteins, cell membrane proteins (e.g., integrins), and cytoskeletal proteins, whose coordinated interactions lead to morphological changes and stable adhesion of osteoblasts [180,181].

It has been shown that the physical properties of biomaterials, such as surface morphology, charge, and pore size—are critical factors influencing osteoblast adhesion [182,183]. Specifically, surface roughness and micro-to nanoscale structures can increase the contact area between osteoblasts and the implant surface, thereby enhancing cell adhesion [[184], [185], [186], [187], [188]]. This enhancement is primarily attributed to the provision of additional anchoring sites for cell attachment [189]. Biggs et al. observed that cells cultured on nanostructured substrates exhibited more extensive pseudopodial extensions and a well-aligned actin cytoskeleton compared to those on nanosmooth surfaces, and optimal adhesion was associated with the presence of nanoscale pits [190]. Furthermore, the presence of surface pores offers additional attachment points that facilitate osteoblast adhesion and spreading [191]. Variations in the spatial distribution of adhesion sites can also influence the binding of ECM proteins to specific integrins, thereby modulating the level of osteoblast adhesion. For example, when the distance between integrin-binding sites and ECM ligands is less than 54 nm, the formation of adhesion protein complexes is promoted [192]. In the context of tissue regeneration engineering, improving osteoblast adhesion can be achieved not only by rationally designing physical features such as surface morphology and pore size, but also by functionalizing the implant surface with biological coatings (e.g., collagen) and growth factors (e.g., BMP-2 and TGF-β) to enhance protein and cell attachment [[193], [194], [195]].

The osteoblast adhesion process is mediated by several important signaling pathways, among which the integrin signaling pathway plays an important role [196,197]. Integrin-mediated adhesion is achieved through receptor-ligand interactions [198], whereby osteoblasts bind to the matrix surface via integrins and subsequently activate downstream signaling molecules such as focal adhesion kinase (FAK) and Src family kinases, which promotes cell survival, migration, and differentiation, thereby reinforcing osteoblast adhesion [199]. Verrier et al. reported that proteins such as fibronectin and OPG possess arginine-glycine-aspartate (RGD) peptide motifs, which enable their binding to integrins [200]. Similarly, Zhao et al. found that COL1, a major ECM component, can also bind to integrin receptors [201], which induces reorganization of the intracellular actin cytoskeleton, regulating cell shape and morphology and promoting adhesion [202]. In addition to integrin signaling, several other pathways significantly contribute to osteoblast adhesion. The PI3K/Akt pathway supports cell survival, inhibits apoptosis, and enhances the expression of osteoblast-specific genes, thereby strengthening adhesion [203,204]. The MAPK pathway, particularly the ERK1/2 cascade, is activated in response to mechanical stimuli and promotes cell proliferation and differentiation, both of which are vital for adhesion and subsequent function [205,206]. Furthermore, integrin activation can upregulate the Wnt signaling pathway, leading to nuclear translocation of β-catenin and increased expression of osteogenic transcription factors such as RUNX2 and Osterix, which are essential for maintaining osteoblast adhesion and functionality [207,208]. Collectively, these signaling pathways are closely interconnected in regulating osteoblast adhesion and associated biological responses, working synergistically to promote bone regeneration.

#### 4.1.2 Osteoblast differentiation

Osteoblast differentiation is a temporally regulated and dynamic process involving the transformation of osteoblast precursors and MSCs into mature osteoblasts. This process is complex and tightly regulated by multiple signaling and environmental factors [209]. MSCs possess multipotent differentiation potential and are the primary cellular source for osteoblast lineage commitment [210]. After their migration and adhesion to the implant surface, MSCs begin to differentiate toward osteoblasts, leading to the production of bone matrix and the formation of mature bone tissue [211]. The efficiency of osteoblast differentiation directly influences the quality and density of the newly formed bone, thereby affecting the strength and stability of the bond between the implant and surrounding bone [212]. Therefore, the regulation of osteoblast differentiation has become a major focus in bone tissue repair research, particularly in bone defect healing and osseointegration [213].

The influence of bone implant surface morphology on osteoblast differentiation is complex, involving a combination of biophysical, chemical, and biological mechanisms [214]. Surface roughness and micromorphology can significantly increase the contact interface between osteoblasts and the implant material, thereby enhancing cell adhesion and altering cell shape and behavior in ways that promote osteogenic differentiation [215]. Nano- and micron-scale surface architectures are particularly effective in mimicking the natural ECM and provide a microenvironment that closely resembles physiological conditions and supports osteoblast differentiation [214,216]. The surface design of titanium implants often imitates the resorption pits created by osteoclasts during bone remodeling, incorporating specific roughness and complex submicron- and nanoscale features that facilitate in vitro osteoblast differentiation and in vivo bone formation [[217], [218], [219]]. Variations in surface roughness can exert differential effects on osteoblast differentiation. For instance, Deng et al. reported that composites with medium surface roughness enhanced calcium nodule formation, increased alkaline phosphatase (ALP) activity, and improved osteogenic differentiation [220]. In a related study, Cunha et al. demonstrated that laser-generated nanotextured surfaces were more effective than polished or micropillar-textured surfaces in promoting matrix mineralization and bone-like nodule formation [122]. "Moreover, appropriate surface porosity and pore size allow osteoblasts to grow and migrate within the scaffold, while also enhancing cell-matrix interactions, thereby promoting cellular activity and osteogenic potential [221]. In addition, Chen et al. found that, during osteogenic differentiation, the size of adherent cells is more important than cell number, emphasizing the importance of cell morphology in promoting osteoblast function and differentiation efficiency [222], highlighting the potential of modulating cell morphology in enhancing osteogenic outcomes.

The effects of implant surface topography on osteoblast differentiation is a complex process involving several key signaling pathways and regulatory mechanisms, including the Wnt/β-catenin pathway, non-canonical Wnt signaling, integrin-FAK signaling, and the BMP signaling cascade. First, micro- and nano-scaled surface morphologies can activate the Wnt/β-catenin signaling pathway by interacting with N-cadherin and β-catenin on the cell membrane, thereby upregulating downstream osteogenic genes such as TCF/LEF and β-catenin itself [62,223,224]. Additionally, micro- and nano-structured topographies can influence the interaction between Frizzled (FZD) receptors and LRP5/6 co-receptors on the cell membrane, leading to Wnt pathway activation. Among these, FZD4 and FZD6 are the most significantly regulated transcriptional components in response to nano-topographies [225,226]. Second, the non-canonical Wnt signaling pathway, through the Wnt/planar cell polarity (PCP) and Wnt/Ca^2+^ branches, modulates cellular behavior associated with skeletal remodeling and bone adaptation [227,228]. Wnt11 and Wnt5a, the key ligands in this pathway, have been shown to regulate both mRNA and protein expression levels of osteogenic genes, playing an important role in promoting osteoblast differentiation in response to micro- and nano-structured surfaces [229,230]. Third, the integrin-FAK signaling axis mediates topographical sensing by interacting with the extracellular matrix, thereby initiating intracellular signaling cascades that promote osteogenic gene expression. Xie et al. demonstrated that the micro- and nano-morphology of graphene substrates activates the integrin/FAK pathway, leading to the formation of phosphorylated FAK (FAK-pY397), recruitment of Talin and Vinculin, activation of SMAD1/5/8, and cytoskeletal remodeling via the RhoA/ROCK pathway. These mechanisms collectively enhance RUNX2 activity and drive osteoblast differentiation and maturation [231]. Similarly, Hwang et al. reported that nanoscale features modulate the nuclear translocation of TAZ via activation of Rho GTPases and MAPK family kinases (JNK, ERK) through the integrin/FAK pathway. TAZ cooperates with RUNX2 in the nucleus to further stimulate the transcription of osteogenic genes, thereby facilitating osteoblast differentiation [[232], [233], [234]]. Lastly, the BMP signaling pathway also plays a pivotal role in mediating the effects of implant topography on osteoblast differentiation. Micro- and nano-textured surfaces of Ti and Ti-6Al-4V alloys have been shown to enhance osteoblast secretion of BMP-2, which exerts paracrine effects on surrounding cells and promotes further differentiation [230,235]. BMPs, acting through the canonical SMAD-dependent pathway, induce phosphorylation of SMAD1/5/8, which subsequently forms complexes with SMAD4 to initiate the transcription of osteogenesis-related genes such as Sp7/OSX and YAP, thereby contributing to bone formation [236,237]. In summary, implant surface topography modulates osteoblast differentiation through a coordinated network of signaling pathways. Optimization of these topographical features holds significant potential to enhance osteogenesis and improve bone regeneration outcomes.

#### 4.1.3 Interactions between osteoblasts and multiple cell types

The morphology of bone regeneration implants has a significant impact on the interactions between osteoblasts and other cell types, including osteoclasts, vascular endothelial cells, neural cells, and immune cells. These interactions are essential in the bone remodeling process and primarily function by enhancing physical cell-cell contact and modulating intercellular signaling.

The synergistic interaction between osteoblasts and osteoclasts at various stages of osteogenesis is essential for lamellar bone formation, bone remodeling, and healing during the later phases of bone regeneration [238]. The dynamic balance between osteoblast and osteoclast activity is central to the regulation of bone mass and exhibits significant spatiotemporal specificity [239]. Implant surface morphology also significantly influences osteoblast-osteoclast interactions by modulating cell adhesion and signaling pathways. For instance, different surface features, such as nanoscale roughness or defined microstructures, can affect the adhesion properties and morphology of both osteoblasts and osteoclasts [240]. Typically, rougher surfaces enhance osteoblast adhesion and spreading, while osteoclasts tend to display greater activity on smoother or specifically microstructured surfaces [241]. Furthermore, surface-induced morphological changes influence intercellular signaling mechanisms [242]. Direct interactions between osteoblasts and osteoclasts mediate mutual regulation of their differentiation and survival through bidirectional signaling pathways such as EFNB2-EPHB4, FasL-Fas or SEMA3A-NRP1 [[243], [244], [245], [246], [247]]. In addition, osteoblasts secrete various molecules, including M-CSF, RANKL/OPG, WNT5A and WNT16, which modulate osteoclast differentiation and development [[248], [249], [250], [251], [252]]. Conversely, osteoclasts can also influence osteoblast formation and differentiation through the secretion of soluble factors, including S1P, SEMA4D, CTHRC1 and C3 [[253], [254], [255], [256]].

The influence of implant surface morphology on osteoblast-vascular endothelial cell interactions is mainly mediated through cell signaling pathways, ECM components, and mechanical stimuli [257]. These interactions are regulated by several signaling pathways, including Wnt/β-catenin, Notch and VEGF signaling [258,259]. Osteoblasts secrete VEGF to facilitate angiogenesis, while endothelial cells reciprocally support osteoblast function through the release of cytokines [213]. Ramasamy et al. demonstrated that H-type vascular endothelial cells enhance bone regeneration via the Notch pathway by secreting factors that stimulate the proliferation and differentiation of bone progenitor cells [260,261]. Furthermore, endothelial cell-derived exosomes contribute to osteogenic differentiation by reprogramming genes within the TGFB1 gene family and upregulating key osteogenic transcription factors, including Osterix (also known as Sp7) and RUNX2 [262]. In addition to these biochemical signals, implant surface morphology influences osteoblast-endothelial cell interactions through modifications in ECM formation and organization. The composition and structural properties of the ECM play a critical role in regulating the functions of both osteoblasts and endothelial cells, as well as their mutual interactions [[263], [264], [265]]. Moreover, the physical characteristics of the implant generate mechanical stimuli that further modulate the coordinated responses of these cell types, particularly affecting their adhesion, migration, and differentiation [266,267].

The morphology of bone regeneration implants significantly influences the interaction between osteoblasts and neuronal cells [268]. This interaction is essential for bone healing and tissue regeneration, as neuronal cells contribute not only to sensory perception and signaling but also to various physiological functions related to bone development and repair [269]. The mechanisms through which implant morphology affects osteoblast-neuronal cell interactions are primarily mediated by the ECM, signaling pathways, and mechanical stimulation [270]. The ECM provides structural support for cell growth and serves as a foundation for cell-cell communication. Its composition and architecture have a substantial impact on the function of both osteoblasts and neuronal cells [271,272]. Osteoblasts and neuronal cells regulate each other's functions via the reciprocal secretion of cytokines and growth factors, including brain-derived neurotrophic factor (BDNF) and nerve growth factor (NGF), which coordinate cell survival, proliferation, and differentiation [273,274]. Okubo et al. reported that sympathetic neuronal cells promote osteoblast differentiation, while osteoblasts, in turn, enhance neuronal differentiation [275]. Additionally, Silva et al. demonstrated that dorsal root ganglion (DRG) neurons promote osteogenesis by regulating the expression of Cx43 and N-cadherin, as well as through activation of the canonical β-catenin/Wnt signaling pathway [276]. He et al. further showed that the osteogenesis-related transcription factor Osterix acts in synergy with Sox10 to regulate central nervous system myelin formation and regeneration, highlighting a shared developmental pathway between oligodendrocytes and osteoblasts [277]. Moreover, the physical morphology of implants generates mechanical stimuli that influence the coordinated behavior of osteoblasts and neuronal cells, thereby modulating their synergistic interactions during bone regeneration [278,279].

The morphology of bone regeneration implants has a significant impact on the interaction between osteoblasts and immune cells, a process that plays a key role in bone healing and tissue regeneration. Implant morphology contributes to the regulation of osteoblast-immune cell interactions through multiple mechanisms [268]. One primary mechanism involves the reciprocal regulation of these cells via cytokine secretion. Osteoblasts and immune cells produce various cytokines, such as IL-6, TNF-α, and TGF-β, which not only modulate immune responses but also promote osteoblast proliferation and differentiation, thereby maintaining homeostasis within the bone microenvironment [280,281]. Wang et al. emphasized the bidirectional communication between the immune and skeletal systems, highlighting the roles of pro-inflammatory cytokines such as TNF-α and IL-6 in both immune regulation and bone metabolism [280,282]. Further evidence of this crosstalk includes findings that depletion of CXCL12 in osteoblasts reduces the population of B-lymphoid progenitor cells in the bone marrow [283] and that expression of the Notch ligand Delta-like 4 by osteoblasts supports T-progenitor cell development [284]. Additionally, osteoblast-derived IL-7 has been shown to be essential for the maintenance of common lymphoid progenitor cells, as demonstrated in osteoblast-specific IL-7-deficient mice [285]. The influence of immune cells on osteoblasts has also been documented. For example, Shono et al. reported that activation of CD4^+^ T cells inhibits osteoblastic bone formation in vivo [286], while Maximiano et al. found that mast cell-mediated factors alter osteoblast proliferation, morphology, and cytoskeletal organization and impair ALP activity and bone sialoprotein expression [287]. In addition to biochemical interactions, the ECM formed through implant-cell interactions serves as a structural platform for osteoblast-immune cell communication. The composition and architecture of the ECM directly influence immune cell migration and function, thereby affecting osteoblast activity [288,289]. Finally, the mechanical stimulation generated by implant morphology further modulates the synergistic behavior of osteoblasts and immune cells, contributing to the overall regulation of bone regeneration [279,290].

#### 4.1.4 Osteoblast apoptosis

The morphology of bone regeneration implants has a significant impact on osteoblast apoptosis, a process that plays a key role in bone formation and reconstruction. The balance between osteoblast survival and apoptosis directly influences bone quality, and the surface characteristics of implants modulate this balance by affecting osteoblast responses to both internal and external stimuli.

The surface roughness and microstructure of implants are key determinants of osteoblast attachment and viability [291]. It has been shown that a moderately rough surface can promote osteoblast survival and reduce apoptosis, whereas smoother surfaces may result in suboptimal osteoblast attachment, thereby increasing apoptosis [292]. In addition, the porosity and pore size of implants are essential factors influencing osteoblast behavior. An optimal pore structure can improve the delivery of nutrients and oxygen, reduce hypoxia-induced oxidative stress in osteoblasts, and promote cell proliferation and survival, which can ultimately lead to a decrease in apoptotic activity [293]. Kulkarni et al. demonstrated that osteoblasts achieved optimal osseointegration on titanium nanotubes with a smaller diameter (15 nm), whereas nanotubes with a larger diameter (100 nm) induced osteoblast apoptosis [294].

### 4.2 Osteoclasts

**Fig. 4:**
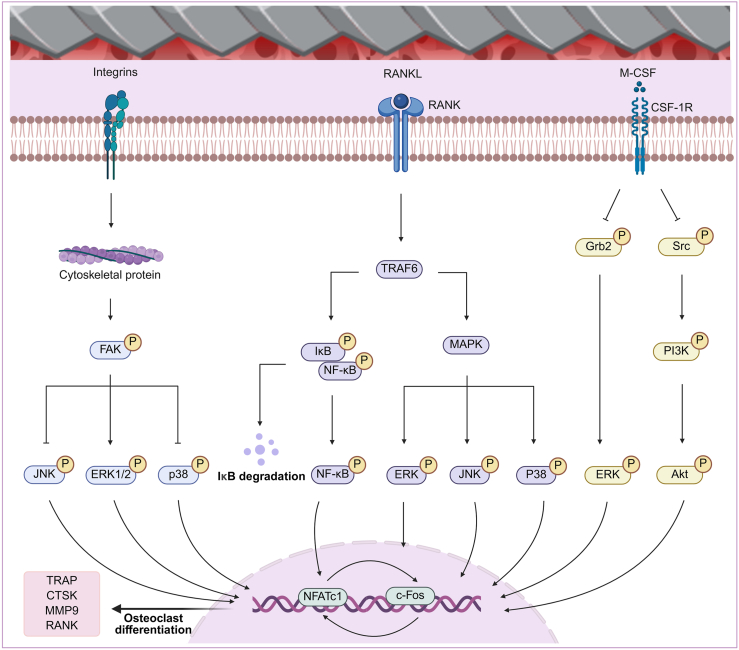
Mechanisms of osteoclast signaling pathways in the implant-bone microenvironment. Created in BioRender. Abbreviations: FAK: Focal adhesion kinase; JNK: c-Jun N-terminal kinase; RANK: Receptor activator of nuclear factor kappa-B; RANKL: Receptor activator of nuclear factor kappa-B ligand; TRAF6: TNF receptor-associated factor 6; MAPK: Mitogen-activated protein kinase; M-CSF: Macrophage colony-stimulating factor; CSF-1R: Colony stimulating factor 1 receptor; PI3K: Phosphoinositol-3 kinase; Akt: Protein kinase B; TRAP: Tartrate-resistant acid phosphatase; CTSK: Cathepsin K; MMP9: Matrix metalloproteinase 9; NFATc1: Nuclear factor of activated T-cells, cytoplasmic, calcineurin-dependent 1.

Optimal implant surfaces play an important role in maintaining bone homeostasis by simultaneously promoting osteoblast differentiation and moderately suppressing excessive osteoclast activity. Surface morphology directly affects net bone accumulation during osseointegration by modulating interactions between osteoclasts and the implant surface [126]. Since osteoclast biological behaviors, including adhesion, differentiation, cellular interactions, and apoptosis, are essential for bone tissue regeneration and remodeling, implant surface topography serves as a key modulator of these cellular processes [295] (Fig. 4).

#### 4.2.1 Osteoclast adhesion

Osteoclasts possess the ability to adhere to and sense their microenvironment, which can in turn regulate cellular activity based on various physiological properties, including mechanical, biomaterial and topographical elements [296]. The bone resorption function of osteoclasts is dependent on their tight adhesion to the bone surface, which is mediated primarily through an actin-rich integrin adhesion structure called the seal zone. The formation and turnover of this structure are highly sensitive to the local environment, including surface chemistry, adhesive properties, and physical characteristics such as microtopography [297]. Notably, three-dimensional sealing zone structures tend to conform to surface geometries, and their migration is often constrained by local topographical barriers [128,298]. During bone remodeling, osteoclasts initially adhere to the bone surface through peduncles, which are specialized dynamic adhesive structures that enable environmental sensing and contribute to cell adhesion, migration, and matrix degradation. These peduncles not only serve as the basic units of the sealing zone but also actively respond to extracellular environmental properties, which are essential for effective bone resorption [[299], [300], [301], [302], [303]]. In support of this, Costa-Rodrigues et al. reported that exposure to nanoscale hydroxyapatite (HA) crystals reduced the differentiation of the mononuclear osteoclast precursor cell line RAW264.7 into mature osteoclasts, suggesting that the adhesion structure of the sealing zone, particularly the pedunculated vesicle, is highly sensitive to the properties of the surface [296,304,305]. Michal et al. also investigated the effect of specific chemical and physical bone surface characteristics on osteoclast adhesion, particularly the sealing ring, and reported that the sealing ring formed preferentially around surface protrusions with transverse dimensions of several micrometers and a height of about 1 μm. In addition, the sealing ring was directly placed over the resorption pit, demonstrating its compatibility with the bone surface during resorption [306].

The osteoclast adhesion process is regulated by several important signaling pathways, with integrin signaling playing a central role. Integrins are adhesion molecules that mediate cell adhesion by recognizing surface morphology, and changes in the three-dimensional morphology of implants can have significant regulatory effects on cell attachment [[107], [108], [109],^307^]. Of particular importance is integrin α_v_β_3_, which is the main form of integrin in osteoclasts and is responsible for signaling through the cytoskeleton and tyrosine phosphorylation cascades upon binding to ECM proteins [308]. Moreover, integrin α_v_β_3_ is abundant in pedunculated vesicles, can recognize ECM ligands and regulate osteoclast interactions with the ECM to facilitate osteoclast attachment to the bone surface, leading to subsequent signaling pathways that promote bone resorption. The strength of osteoclast adhesion and the process of bone resorption are modulated by integrin α_v_β_3_ through its binding to specific surface features [117,[309], [310], [311], [312], [313]]. Wang et al. demonstrated that matrix stiffness influences osteoclast fate through two parallel mechanisms: RhoA-ROCK2-YAP-mediated mechanotransduction involving integrins, and NF-κB-mediated biochemical signaling involving RANK. Activation of the NF-κB pathway via RANK promotes the transcription of NFATC1, a key osteoclastogenic transcription factor, thereby regulating the expression of osteoclast-related proteins such as TRAP and CTSK [314]. Kong et al. also reported that osteoclasts must adhere to and migrate along the bone matrix to facilitate erosion. In this process, integrins regulate the formation of pedunculated vesicles and cytoskeletal dynamics, working in conjunction with other molecules such as c-Cbl and p130 [305]. In addition to integrin signaling, other key molecular pathways influence osteoclast adhesion. For instance, Dufrancais et al. found that Moesin, a cytoskeletal junction protein from the Ezrin/Radixin/Moesin (ERM) family, is activated during osteoclast maturation and plays a pivotal role in osteoclast function. Moesin depletion reduces the attachment of the cell membrane to the bone cortex and promotes the formation of tunneling nanotubes. Additionally, Moesin controls bone resorption by regulating sealing zone formation through the integrin-β3/RhoA/SLK signaling pathway, highlighting its importance in osteoclast adhesion and function [315].

#### 4.2.2 Osteoclast differentiation

Various surface microstructures with differing geometrical and dimensional morphologies have been shown to modulate osteoclast differentiation; however, there remains no consensus regarding the direction of this modulation. Several studies have reported that surface microstructures exert an inhibitory effect on osteoclast differentiation. For instance, Akasaka et al. compared the effects of micrometer- and sub-micrometer-scale columns, grooves, and pores, each designed with a 1:1 spacing ratio, on osteoclastogenesis in RAW264.7 cells, and found that columns with a diameter of 500 nm and height of 2 μm promoted the formation of osteoclast-like cells, whereas smaller columns with a diameter of 100 nm and height of 200 nm reduced their formation [316,317]. Moreover, He et al. proposed that titanium surface nanotopography with a nanotube diameter of approximately 30 nm inhibits osteoclast differentiation by downregulating the expression of integrin β1 and phosphorylated FAK (FAKpY397), thereby suppressing the phosphorylation of JNK and p38 and disrupting MAPK cascade signaling. This inhibitory effect may be attributed to the inability of porous nanostructures to provide adequate adhesion sites, which hinders osteoclast migration and fusion [63]. In line with this, Wang et al. employed photolithography to construct concentric microlayers with a width and depth of 10 μm Consistent with these findings, Wang et al. employed photolithography to fabricate concentric microlayers with a width and depth of 10 μm, demonstrating that such surface features guided cell migration and altered cell morphology. Compared with osteoclasts cultured on smooth titanium surfaces, those grown on microgrooved surfaces exhibited significantly reduced osteoclastogenesis [318]. Building upon this, Li et al. combined photolithography with a fusion casting technique to fabricate an osteon-like concentric microgrooved (OCM) surface that mimics the structural dimensions of a natural bone unit (200 μm outer diameter, 20 μm groove width, 20 μm groove depth, and 5 μm spacing between adjacent grooves). In comparison with parallel linear microgroove (PLM) surfaces (unit side length 200 μm; groove width and depth identical to OCM) and flat planar surfaces, the OCM design significantly inhibited osteoclast differentiation in RAW264.7 cells. This was evidenced by suppression of the M-CSF and RANK-NF-κB signaling pathways, downregulation of osteoclast-specific functional genes (TRAP, CTSK, and MMP9), reduced TRAP activity, and a decrease in the formation of TRAP-positive multinucleated osteoclasts [319].

In contrast to these findings, several studies have suggested that surface microstructures can promote osteoclast differentiation. For example, rough metal implant surfaces have been shown to enhance osteoclast activity, while smoother surfaces are associated with reduced differentiation capacity [299]. Sommer et al. compared rough surfaces introduced by sandblasting with polished smooth surfaces and found that, regardless of substrate composition, osteoclastogenesis was consistently higher on rough surfaces [320]. Jeong et al. further explored this by developing micropatterned surfaces featuring parallel grooves 2 μm in width and 1 μm in depth, with varying spacing of 1, 5, or 10 μm. They observed that the grooves with the smallest spacing (1 μm) significantly enhanced osteoclastogenesis in rat bone marrow-derived cells compared to wider grooves. The authors proposed that the 1 μm pattern modulates integrin-mediated signaling to reduce myosin II tension, thereby increasing peduncle stability and promoting osteoclast differentiation [296]. Costa et al. examined osteoclast activity on HA coatings with different isotropic topographies containing sub-micron and micron-scale features. Their results showed that anti-tartrate hydrochloride acid phosphatase activity, a marker of osteoclast differentiation, was higher on the smoother surface (Ra ∼1 μm) than on the more complex microrough surface (Ra ∼2 μm) [321]. Despite progress in understanding the mechanisms by which topographical features regulate osteoclast behavior, further research is needed to clarify the specific effects of surface microstructures on osteoclastogenesis and to achieve a unified interpretation of their regulatory roles.

#### 4.2.3 Interactions between osteoclasts and multiple cell types

Coordination between osteoclasts and various other cell types is essential for maintaining the osseointegration of bone implants. In particular, the crosstalk between osteoclasts and osteoblasts is strongly influenced by surface topography. For example, Bighetti-Trevisan et al. demonstrated that osteoclasts can inhibit osteoblast function on titanium surfaces. However, when titanium nanotopographies were generated using H_2_SO_4_/H_2_O_2_ treatment, the resulting surface modifications provided a protective effect for osteoblasts. This protective mechanism was attributed to the ability of the nanotopography to inhibit excessive accumulation of H3K27me3 in the promoter regions of RUNX2 and Alp, thereby modulating histone methylation and reducing suppression of gene expression. As a result, osteoclast-induced inhibition of osteoblast differentiation and function was attenuated [[322], [323], [324]]. In addition to these effects, titanium dioxide nanotubes (Ti-NTs) have been found to inhibit osteoclast formation and function in a diameter-dependent manner. Specifically, Ti-NTs with a diameter of 90 nm suppressed the expression of integrin ανβ3 in osteoclast precursor cells, thereby inhibiting osteoclastogenesis. These properties make such surfaces particularly beneficial for potential applications in the treatment of osteoporosis [325].

Osteoclasts also regulate the local bone microenvironment through the secretion of various cytokines and extracellular vesicles, which serve as important modulators of bone homeostasis [326,327]. Among these secreted molecules, osteoclastogenic cytokines, often referred to as clastokines, act as coupling factors that influence osteoblast behavior and recruitment [328,329]. Certain clastokines, including BMP6, CT-1, Cthrc1, HGF, Slit3, Sphk1 and Wnt10b, are known to promote osteoblast recruitment and enhance matrix deposition and mineralization [[329], [330], [331], [332], [333], [334], [335]]. In contrast, other osteoclast-derived factors, such as SC and Sema4d, have been shown to exert inhibitory effects on bone formation [254]. Surface nanotopography has also been shown to modulate the expression of these osteoclast-derived signaling molecules. Specifically, nanotopographical stimulation was found to increase the expression of osteoclastogenic cytokines that support bone formation while simultaneously suppressing the expression of cytokines that promote bone resorption [336]. Among the osteoclasts formed on the surface of TiO_2_ nanotubes with a diameter of 30 nm, a notable upregulation of the Slit3 gene was observed. SLIT3 is considered a protective factor in bone remodeling, as it contributes to the coordinated regulation of bone formation and resorption [63].

#### 4.2.4 Osteoclast apoptosis

The physical characteristics of implant surface topography, including roughness and micro- and nanostructures, can impact osteoclast survival and apoptosis. Differentiated mature multinucleated osteoclasts attach to bone surfaces and resorb bone matrix, and upon reaching a certain resorption depth, these osteoclasts undergo apoptosis, followed by recruitment of osteoblasts to initiate bone reconstruction at the resorption site [337]. The prolonged survival of osteoclasts contributes to the development of skeletal disorders [338]. For instance, in osteoporosis, excessive osteoclast activity extends their survival time, thereby accelerating pathological bone loss [339]. Several studies have examined strategies to modulate osteoclast apoptosis through surface modifications. Ko et al. found that surface-porous titanium implants improved osseointegration in osteoporotic dogs following bilateral oophorectomy [340]. Similarly, Zhao et al. demonstrated the efficacy of micro/nano-structured calcium phosphate bioceramics in repairing osteoporotic bone defects [341]. Furthermore, micro- and nanoscale surface features can indirectly affect osteoclast viability by modulating interactions between osteoclasts and osteoblasts [342]. Therefore, the reasonable design of implant surface topography offers a promising strategy to promote osteoclast apoptosis, reduce their survival time, and mitigate bone loss in vivo.

### 4.3 Macrophages

**Fig. 5:**
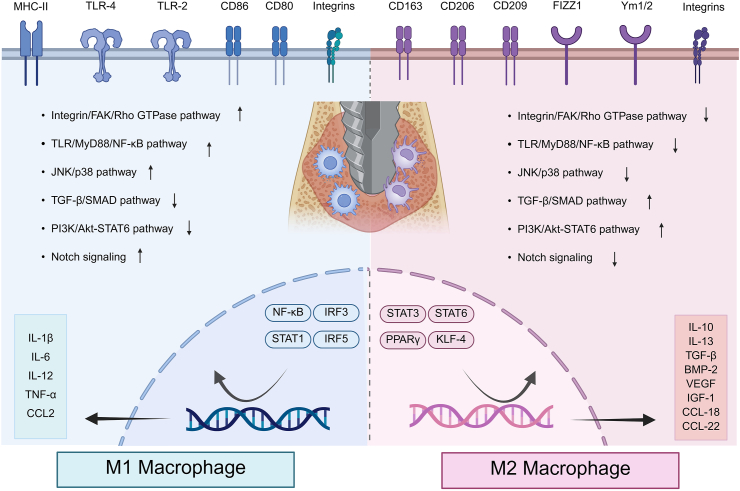
Mechanisms of macrophage signaling pathways in the implant-bone microenvironment. Created in BioRender. Abbreviations: MHC-II: Major histocompatibility complex, class II; TLR: Toll-like receptor; CD86: Cluster of differentiation 86; FIZZ1: Fibrinogen-like protein 1; Ym1/2: Macrophage inflammatory protein 3, neutrophil gelatinase-associated lipocalin; FAK: Focal adhesion kinase; SMAD: Small mother against decapentaplegic; PI3K: Phosphoinositol-3 kinase; Akt: Protein kinase B; MyD88: Myeloid differentiation primary response 88; STAT: Signal transducer and activator of transcription; IRF: Interferon regulatory factors; KLF-4: Krüppel-like factor 4; IL-10: Interleukin 10; BMP-2: Bone morphogenetic protein 2; VEGF: Vascular Endothelial Growth Factor; IGF-1: Insulin-like Growth Factor 1; CCL-18: Chemokine (C-C Motif) Ligand 18.

Recent studies have shown that the surface morphology of implants has a significant effect on macrophage behavior. As key components of the immune system, macrophages play a direct role in modulating the host immune response to implants and significantly affect bone regeneration outcomes [[343], [344], [345]]. Surface topographical features, including nanoscale structures and surface roughness, have been shown to regulate macrophage adhesion and polarization, and to mediate cell-cell interactions via several signaling pathways, such as PI3K/Akt, MAPK and NF-κB [[346], [347], [348]]. Additionally, the surface morphology of bone regeneration implants has been reported to impact macrophage apoptosis [349]. Therefore, a comprehensive understanding of macrophage-implant surface interactions is essential for optimizing implant design and enhancing bone regeneration (Fig. 5).

#### 4.3.1 Macrophage adhesion

Macrophages are central to the host immune response, and their adhesion behavior on implants determines implant biocompatibility and long-term stability [350]. Surface topography, including nanostructures and surface roughness, can significantly influence macrophage adhesion [351,352]. Several studies have demonstrated that micro- and nanoscale roughness markedly enhances macrophage adhesion [353,354]. Similarly, nanostructured surfaces such as nanowires and nanopores have been found to improve macrophage adhesion behavior [343,355]. For instance, Chen et al. [355] reported that macrophages exhibited stronger adhesion and proliferation on nanoporous surfaces with larger pore sizes (200 nm), while smaller pore sizes (50 nm and 100 nm) were less favorable for cell adhesion [356]. In contrast, a study by Gulati et al. [357] showed that nanoporous surfaces with pore sizes of 60–70 nm significantly reduced macrophage adhesion and proliferation, suggesting a strong immunomodulatory effect compared to other surfaces, including smooth, rough, or nanotubes with different pore sizes. The mechanisms by which surface morphology regulates macrophage adhesion behavior involve several interconnected pathways, such as integrin-mediated signaling, ECM protein adsorption, and dynamic cytoskeletal rearrangement [349,358]. Macrophages initially recognize and bind to ECM proteins such as fibronectin and laminin through integrin receptors, including α_v_β_3_ and α5β1, which subsequently activate downstream signaling cascades such as PI3K/Akt and FAK pathways [[359], [360], [361], [362], [363], [364], [365]] to promote cytoskeletal reorganization and adhesion [366]. Furthermore, micro- and nanoscale surface features significantly influence ECM protein adsorption [367]. Rough and nanostructured surfaces can increase the adsorption of fibronectin and laminin, thereby facilitating stronger macrophage attachment [349,368]. In addition, the dynamic rearrangement of the cytoskeleton is an essential determinant of macrophage adhesion [369]. Nanostructured surfaces activate small Rho GTPase family proteins, including RhoA, Cdc42, and Rap1, which in turn regulate cytoskeletal remodeling and enhance the adhesion capability of macrophages [343,358,370].

#### 4.3.2 Macrophage polarization

Macrophages, due to their inherent plasticity and capacity for polarization, exhibit dynamic functional shifts at different stages of bone healing and play a pivotal role in regulating the outcome of bone regeneration [371]. These cells can polarize into either pro-inflammatory M1 or anti-inflammatory M2 phenotypes [372]. M1-type macrophages are primarily involved in initiating inflammatory responses and secrete pro-inflammatory cytokines such as IL-1β, IL-6, IL-12, TNF-α, and CCL2. This phenotype is regulated by transcription factors, including NF-κB, STAT1, IRF3, and IRF5. In contrast, M2-type macrophages contribute to anti-inflammatory processes and tissue repair by producing factors such as IL-10, IL-13, TGF-β, BMP-2, VEGF, IGF-1, CCL-18, and CCL-22. The polarization toward the M2 phenotype is controlled by transcription factors such as STAT3, STAT6, PPARγ, KLF-4, and IRF4 [85,373,374]. It is recognized that implant surface morphology can regulate the direction of macrophage polarization [349]. One strategic approach to minimizing acute inflammation involves utilizing the phagocytic capacity of M1 macrophages to clear debris and initiate an inflammatory microenvironment that supports subsequent tissue remodeling [375], while another approach focuses on promoting M2 macrophage polarization due to their role in osteoinduction and bone formation [376,377]. Nanostructured surfaces generally promote polarization toward the M2 phenotype, whereas rougher, disordered, or smooth surfaces tend to favor M1-type macrophage activation [378]. Further investigations have demonstrated that nanotube diameter significantly influences macrophage polarization. Specifically, nanotubes with a diameter of 100 nm tend to induce M1 polarization, characterized by elevated secretion of pro-inflammatory mediators such as iNOS, TNF-α, and IL-1β. In contrast, nanotubes with a diameter of 30 nm preferentially promote M2 polarization, enhancing the secretion of anti-inflammatory and pro-regenerative cytokines such as IL-10, VEGF, and BMP-2 [104,379]. Interestingly, some researchers have proposed that a sequential polarization pattern, initial activation of M1 macrophages followed by a transition to the M2 phenotype, may offer optimal conditions for bone regeneration [349,380].

The effect of implant surface topography on macrophage polarization involves multiple complex signaling pathways. Surface features such as roughness and texture directly modulate integrin expression and activation of the integrin-FAK-Rho GTPase signaling axis in macrophages. Rough or structured surfaces increase the contact area between macrophages and the implant, thereby promoting the clustering of integrins (e.g., αMβ2 and α5β1) and subsequent activation of the PI3K/Akt-STAT6 pathway, which plays a critical role in regulating macrophage survival, proliferation, and polarization [[381], [382], [383]]. Integrin engagement may also trigger the MAPK signaling cascade, including ERK, p38, and JNK, which orchestrates a wide range of cellular responses [384,385]. In parallel, specific nanoscale surface features can promote M2 polarization by suppressing NF-κB signaling activity, thereby enhancing the production of anti-inflammatory cytokines such as IL-10 and TGF-β [[385], [386], [387], [388]]. Other regulatory mechanisms have also been identified. For example, Myosin 1F has been shown to facilitate M1 polarization by strengthening intercellular adhesion through the integrin-αVβ3-ILK-Akt-mTOR signaling axis [389,390]. Moreover, Toll-like receptors (TLRs) on macrophages can sense the biological properties of implant surfaces. Upon activation of the TLR-MyD88-NF-κB pathway, downstream signaling cascades are initiated that promote M1 differentiation and enhance the secretion of chemokines, contributing to the recruitment of additional immune cells and modulation of the local immune microenvironment [391,392]. Mechanical cues derived from surface topography also play a role in macrophage polarization by influencing cytoskeletal organization and mechanoresponsive gene expression patterns. For instance, rougher surface morphologies and increased matrix stiffness enhance macrophage adhesion and promote polarization toward the M1 phenotype. In this state, macrophages secrete pro-inflammatory cytokines, including IL-1β, IL-6, IL-12, TNF-α, and CCL2, under the control of transcription factors such as NF-κB, STAT1, IRF3, and IRF5 [358,393]. Conversely, smoother surfaces or lower substrate stiffness can promote M2 polarization through the activation of mechanosensitive channels such as Piezo1. M2 macrophages, regulated by transcription factors including STAT3, STAT6, PPARγ, KLF-4, and IRF4, secrete anti-inflammatory and pro-regenerative mediators such as IL-10, IL-13, TGF-β, BMP-2, VEGF, IGF-1, CCL-18, and CCL-22, thereby facilitating tissue repair and resolution of inflammation [394,393]. In summary, implant surface morphology exerts a substantial influence on macrophage polarization through multiple signaling pathways and mechanotransductive mechanisms. These findings provide a theoretical foundation for the design of biomaterial surfaces that can modulate immune responses to promote favorable outcomes in bone regeneration.

#### 4.3.3 Interactions between macrophages and multiple cell types

The surface morphology of bone regeneration implants plays an important role in mediating interactions between macrophages and other cell types, including vascular endothelial cells, neuronal cells, osteoblasts, and osteoclasts. One of the key effects of implant surface morphology is its ability to modulate macrophage polarization, which in turn influences downstream biological processes. For instance, the anti-inflammatory cytokines and angiogenic factors secreted by M2-type macrophages enhance the proliferation and migration of vascular endothelial cells, thereby facilitating the formation of vascular networks and promoting angiogenesis [[395], [396], [397]]. It has been demonstrated that changes in implant surface morphology can regulate both macrophage recruitment and polarization, indirectly influencing angiogenesis [349,374,398]. In addition to their role in vascularization, macrophages contribute to nerve regeneration and repair through the secretion of neurotrophic factors such as NGF, as well as anti-inflammatory cytokines [399,400]. Surface morphology indirectly affects neuronal function by modulating the macrophage polarization state [349,401,402]. Macrophage polarization also significantly impacts osteoblast activity and function. M1-type macrophages secrete pro-inflammatory cytokines such as TNF-α, IL-1, IL-6, and GM-CSF, which inhibit osteoblast maturation and mineralization, in part by regulating the Notch signaling pathway [372,403,404]. In contrast, M2-type macrophages stimulate osteoblast proliferation and differentiation by releasing signaling molecules, including BMP-2, TGF-β, and PGE2 [372,374,[405], [406], [407], [408]]. As a result, implant surface morphology can influence osteoblast function indirectly by affecting macrophage polarization [409,410]. Furthermore, macrophages play a key role in osteoclast differentiation and maturation [374]. M1-type macrophages are capable of differentiating into osteoclast precursors and promoting osteoclast formation through the secretion of cytokines such as TNF-α, IL-6, and RANKL [[372], [378], [410], [411]]. Implant surface morphology thus indirectly affects osteoclast activity by regulating the polarization state of macrophages [394,412].

#### 4.3.4 Macrophage apoptosis

The surface topography of bone regeneration implants has a significant influence on macrophage apoptosis. For instance, implants with rough or porous surfaces tend to promote macrophage survival, whereas smoother surfaces are often associated with increased rates of apoptosis [349,413,414], potentially because rough surfaces enhance cell-matrix interactions, which facilitate the activation of cell survival signaling pathways [103]. During bone regeneration, the effect of surface topography on macrophage apoptosis is primarily mediated through three mechanisms. First, surface topography alters macrophage morphology and cytoskeletal organization, which subsequently affects mechanotransduction pathways. These structural changes can regulate intracellular signaling cascades involved in cell survival and apoptosis, such as the SIRT6-mediated chemotaxis pathway [349,415,416]. Second, surface morphology influences the polarization state of macrophages, thereby altering their cytokine secretion profiles. M1-type macrophages, which are predominant in pro-inflammatory environments, are generally more prone to apoptosis. In contrast, M2-type macrophages contribute to the resolution of inflammation and reduce apoptotic activity by secreting anti-inflammatory cytokines such as IL-10 and TGF-β [348,376,394,417]. Third, implant surface features can indirectly impact macrophage apoptosis by modulating their interactions with other cell types, including osteoblasts and osteoclasts [348,372,373]. For instance, macrophages are known to participate in bone remodeling by phagocytosing apoptotic osteoblasts, thereby influencing local immune responses and bone homeostasis [[418], [419], [420]].

### 4.4 Regulatory mechanisms underlying the interplay between implant topography and other immune cells

Although macrophages remain the most extensively studied immune cells in implant-mediated immune responses and bone regeneration, growing interest has emerged in elucidating the roles of other immune cells, such as neutrophils, T cells, B cells and DCs. Implant surface morphology can modulate the behavior of these immune cells, and their interactions collectively contribute to shaping the immune microenvironment and ultimately influence the outcomes of bone regeneration. For example, Morandini et al. reported that rough implant surfaces produced by sandblasting and acid etching reduced neutrophil recruitment and the formation of neutrophil extracellular traps (NETs). These surfaces were associated with decreased secretion of pro-inflammatory cytokines and enhanced production of anti-inflammatory cytokines when compared to smooth surfaces. Furthermore, the reduction in NET formation led to diminished recruitment of macrophages, DCs, and T cells, while enhancing the recruitment of MSCs. This shift in cellular dynamics contributed to the attenuation of inflammation and facilitated peri-implant bone formation [421]. A deeper understanding of how implant surface morphology interacts with diverse immune cell types is essential to uncover the complex immunoregulatory mechanisms involved in bone regeneration. Such insights will provide a theoretical foundation for the development of next-generation smart implants that possess tailored immunomodulatory properties.

#### 4.4.1 Neutrophils

The surface topography of bone implants has a significant effect on the behavior and function of neutrophils, which in turn affects the process of bone regeneration [422,423]. It has been shown that surface topography can regulate neutrophil activity and function through several mechanisms, thereby affecting bone metabolism and repair.

Neutrophils play an essential role in the early stages of bone regeneration. As the first immune cells to reach the site of inflammation, they function by capturing and eliminating pathogens through the release of NETs [424,425]. NETs are composed primarily of DNA, granule proteins, and nuclear proteins, which work together to encapsulate and neutralize invading microorganisms [[426], [427], [428], [429]]. Beyond their role in inflammation, neutrophils have been shown to inhibit osteoblast differentiation via direct interaction with osteoclasts and to promote osteoclast-mediated bone resorption through RANKL expression [[430], [431], [432]]. Moreover, neutrophils contribute to bone regeneration by recruiting bone marrow-derived BMSCs through the secretion of cytokines such as IL-8 [[433], [434], [435], [436], [437]], which is mediated by the SDF-1/CXCR4 axis and involves downstream signaling via the PI3K/Akt pathway and β-catenin-regulated cell migration [436,[438], [439], [440]].

Implant surface morphology also modulates neutrophil chemotaxis and polarization [430]. Nanostructured surfaces have been shown to enhance the adsorption of ECM proteins such as fibronectin and laminin, thereby promoting neutrophil adhesion and NET formation [441,442]. These surface features activate neutrophils via integrin-mediated signaling pathways, including PI3K/Akt and MAPK, which regulate NET release [[443], [444], [445]]. However, excessive NET formation may lead to tissue damage and chronic inflammation [424,442,446]. By optimizing surface morphology, it is possible to reduce excessive NET production, thereby mitigating chronic inflammation and improving implant success rates [441,447,448]. Additionally, surface topography influences neutrophil polarization. Rough implant surfaces have been shown to promote polarization toward the N2 phenotype, characterized by increased secretion of SDF-1α, which enhances the recruitment of BMSCs [436,438]. In contrast, smooth surfaces may suppress neutrophil activity and reduce their capacity to attract BMSCs [[225], [430]]. Lastly, surface topography has been reported to influence neutrophil apoptosis, with rough surfaces reducing their apoptosis rate and thereby extending their functional activity at bone regeneration sites [27,430,449].

#### 4.4.2 T lymphocytes

Studies have shown that implant surface topography can influence T lymphocyte activation through multiple mechanisms [450,451]. First, T lymphocytes exhibit higher adhesion capacity and effector activity on surfaces with increased roughness and complex microstructures [[452], [453], [454]]. This enhanced response is attributed to the fact that rough surfaces promote cell attachment and interaction, which in turn facilitates T lymphocyte activation [455,456]. Second, T lymphocyte activation is dependent on antigen presentation and co-stimulatory signals, and alterations in implant surface morphology can optimize the activation of antigen-presenting cells, such as macrophages and DCs, which stimulate T lymphocytes through Fas ligands and cytokines such as IL-2 and IFN-γ [450,[457], [458], [459], [460], [461], [462], [463]]. Moreover, modulating implant surface morphology can influence T lymphocyte responses while simultaneously supporting bone regeneration [451,464], making the design of surface topographies that optimize T cell activation an important focus of research.

#### 4.4.3 B lymphocytes

The surface topography of bone regeneration implants significantly influences the function and behavior of B lymphocytes, primarily through micro- and nanostructural features that regulate their adhesion, proliferation, and differentiation, thereby impacting the overall bone regeneration process [465,466]. First, the micro- and nanoscale structures of the implant surface directly affect the adhesion and proliferation of B lymphocytes [[467], [468], [469]]. Second, surface topography also plays a critical role in modulating B lymphocyte differentiation and functional activity [[469], [470], [471]]. In addition, implant surface morphology can indirectly influence B lymphocyte function by modulating the polarization states of other immune cells [466,469]. For instance, nanoscale surface features have been shown to promote macrophage polarization toward anti-inflammatory and regenerative phenotypes, thereby creating a microenvironment more conducive to B lymphocyte function [[472], [473], [474]]. Furthermore, specific surface morphologies can stimulate B lymphocytes to secrete anti-inflammatory cytokines to further enhance bone regeneration [469]. Mechanistically, these effects may be mediated through the activation of several signaling pathways involved in cell proliferation and differentiation, including PI3K, MAPK/ERK, NF-κB, and Akt pathways [[475], [476], [477]]. Thus, implant surface topography can influence B lymphocyte behavior through multiple direct and indirect mechanisms to affect the bone regeneration process. Future studies are warranted to further elucidate the molecular mechanisms underlying these interactions, with the aim of designing more effective bone regeneration implants.

#### 4.4.4 Dendritic cells

The surface morphology of implants has a significant influence on the function and activity of DCs. First, rough surfaces promote DC aggregation and enhance their activity by increasing the available surface area for cell attachment [478,479]. In particular, moderate surface roughness has been shown to significantly improve DC function [480,481]. Second, variations in surface morphology induce morphological changes in DCs and influence the formation of their extended membrane protrusions [482]. Optimized surface features can enhance the number and structure of these protrusions to improve the cells’ capacity for antigen capture and presentation [483]. The cellular response of DCs to such morphological changes involves the activity of WASP and the Arp2/3 complex, which facilitate the nuclear accumulation of cytosolic phospholipase A2 (cPLA2) and subsequent upregulation of CCR7 expression [484,485]. In addition, implant surface topography modulates DC-matrix interactions, thereby regulating key signaling pathways such as MAPK and TLR4/MYD88/NF-κB, which are involved in DC maturation and cytokine production [486,487]. Collectively, an optimized surface morphology can enhance the antigen-presenting ability of DCs, promote cytokine secretion and facilitate the activation and proliferation of T lymphocytes to strengthen the overall immune response [488,489].

## 5 Topography-based implants for in vivo therapeutics

Topography-based bone implants have been designed and applied for the repair of bone defects under various pathophysiological conditions and at specific implantation time points. These implants have demonstrated favorable outcomes in promoting bone regeneration and repair, even in complex pathological environments such as diabetes mellitus and osteoporosis. The primary design strategy involves modulating the activity of osteoblasts and immune cells within the bone microenvironment through tailored surface morphologies, thereby enhancing the bone healing process (Table 2).

**Table 2:** Topography-based implants for in vivo therapeutics.

In vivo therapeutics	Implant morphological characterization	Topography preparation methods	Types of materials	Animal models	In vivo therapeutic effects	Cells phenotype	Topology-related molecular pathways	Ref.
Critical bone defects	Nanoscale surface morphology of vertically aligned nanofeatures with diameters less than 100 nm	Oxidation and oxalic acid treatment	Titanium	Critical tibial defect model in rats	Improved osseointegration of implants	Macrophage shift towards anti-inflammatory and osteogenic phenotypes	Up-regulation of the expression of osteogenesis-related genes and down-regulation of inflammatory gene expression	[469]
	Nanoscale surface morphology such as nanotubes, with sizes in the range of 1–100 nm and a roughness parameter (Ra) of 341–427 nm	Sandblasting and acid etching	Titanium	Critical femoral defect model in mice	Promotion of osseointegration, increased rate of osseointegration and bone volume	Promotion of osteoblast differentiation	Up-regulation of the expression of osteogenesis-related genes	[490]
	Surface morphology with micron and nanometer roughness and high hydrophilicity	Laser sintering technology, sandblasting, acid etching	Titanium alloy	Critical femoral defect model in New Zealand white rabbits	Facilitation of implant osseointegration	Enhancement of osteoblast differentiation	Up-regulation of the expression of osteogenesis-related genes	[491]
	Fish scale morphology with natural ridge micropatterns	Decellularization and de-collagenization processes	Fish scales	Critical femoral defect model in rats	Promotion of new bone formation and osseointegration	Promotion of osteoblast adhesion, proliferation, and differentiation; Induction of M2 macrophage polarization	Activation of the Wnt/β-catenin signaling pathway, increased expression of osteogenesis-related proteins and increased secretion of anti-inflammatory cytokines	[492]
	Laminar morphologic structure of bionic natural bone	Decellularization, mineralization	Fish scales	Critical cranial defect model in rats	Facilitation of bone defect repair, and more mature neoplastic bone tissue	Facilitation of adhesion, proliferation, and osteogenic differentiation of BMSCs	Up-regulation of osteogenesis-related genes	[493]
Dental implants	Nested nanofiber structure	Electrochemical anodizing, alkali etching	Titanium	Alveolar bone implant models in Beagles	Improvement osseointegration capacity	Promotion of adhesion, survival, and proliferation of BMSCs	Increased expression of osteogenesis-related genes	[494]
Diabetic bone defects	Hydrophilic nano-surfaces	Sandblasting, acid etching, hydrophilic treatment	Titanium	Cranial defect model in diabetic rats	Promotion of new bone formation and improvement of bone healing	M2 macrophage polarization	Inhibition of the activation of the PI3K/Akt/mTOR signaling pathway, increase in the secretion of the anti-inflammatory cytokines and decrease in the production of pro-inflammatory cytokines	[65]
Osteoporotic bone defects	Hydrophilic nano-surfaces	Sandblasting, acid etching, hydrophilic treatment	Titanium	Femoral defect model in osteoporotic rats	Higher bone implant contact rates and torque values	Increased osteoblast activity, decreased osteoclast activity	Activation of PI3K/Akt and Wnt signaling pathways and increased expression of OCN, OPN, BMP-2 and OPG	[67]
	Petaloidal structure with microgrooves, islands and micropores, surface roughness about 0.884 μm	Femtosecond laser etching and sulfonation	PEEK	Femoral defect model in osteoporotic rats	Improvement of implant osseointegration	M2 macrophage polarization	Activation of MET and Ras/TIAM1 signaling pathways and enhancement of anti-inflammatory factor secretion and reduction of pro-inflammatory factor expression	[495]
	Bionic porous structure with randomly distributed holes of different diameters	Hot pressing and sintering	Magnesium alloy	Femoral defect model in osteoporotic rats	Promotion of implant osseointegration	Promotion of migration, proliferation, and differentiation of BMSCs	Activation of Wnt/β-catenin signaling pathway and improvement of the expression levels of Wnt5a, β-catenin and BMP-2	[496]

### 5.1 Critical bone defects

Animal studies have demonstrated that the surface topography of implants plays a key role in the repair of critical bone defects. Firstly, sub-micron to micron-scale surface topographies have been shown to facilitate implant osseointegration in animal models of critical bone defects. Davies et al. [497,498] constructed surface topographies with various features and scales on the surface of customized titanium alloy (Ti_6_Al_4_V) implants using multiple techniques, including polishing (PL), machining (MC), double-acid-etching (DAE), and grit blasting followed by acid etching (GB/AE1 and GB/AE2), with further deposition of nanocrystals (DCD) on some surfaces. These surface morphologies spanned sub-micron to micron scales and specifically included the smallest microscale features in polished and machined surfaces, more complex microscale morphologies in double-acid-etched and sandblast-acid-etched surfaces, and the highest surface roughness and complexity in sandblast-acid-etched variants. These topographical modifications enhanced bone-implant bonding by promoting osteoblast recruitment, migration, adhesion, proliferation, and differentiation, as well as by supporting the formation of mineralized cement lines and collagen fibers on the implant surface. Implants with complex microscale and coarse micrometer-scale features (e.g., GB/AE) exhibited superior osseointegration and interfacial stability under long-term loading conditions in rat femoral critical bone defect models, as demonstrated by enhanced mechanical resistance and histological evidence of improved bone-implant integration.

**Fig. 6:**
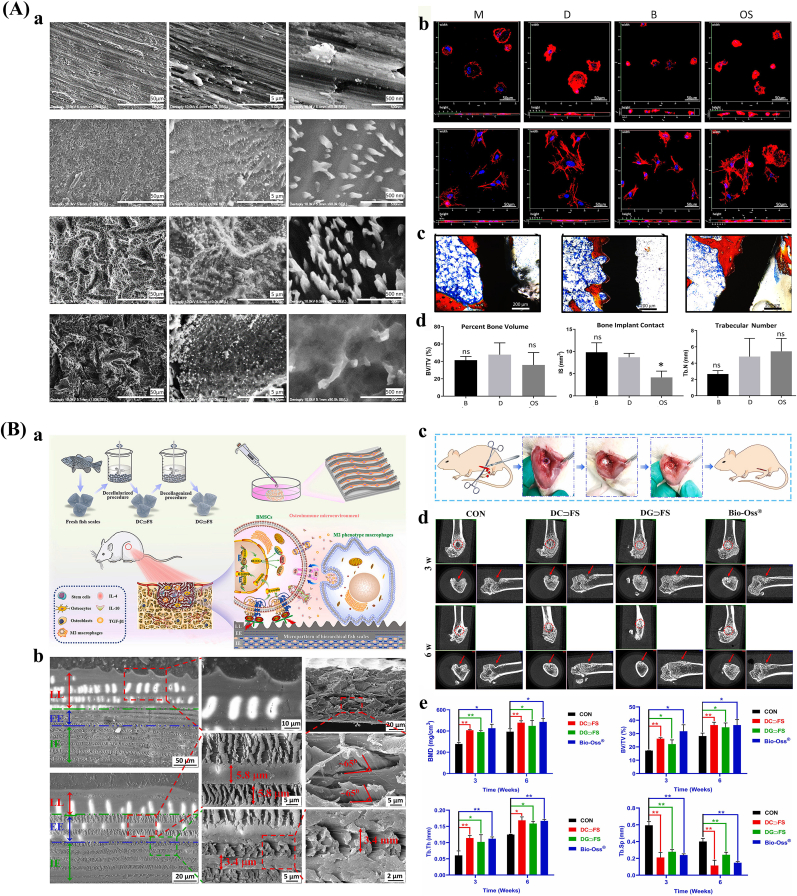
Topology-based implants for in vivo treatment of animal models of critical bone defects. (A) Nanoscale and hybrid micro- and nanocomposite surfaces for intraosseous implants. a. SEM images. b. Confocal images of HMSCs. c. Ground sections of bone-implant interface. d. Bone micromorphometry parameters [469]. (B) Natural micropatterned fish scales combing direct osteogenesis and osteoimmunomodulatory functions for enhancing bone regeneration. a. Schematic illustration. b. SEM images. c. Critical bone defect modeling and implant filling. d. Micro-CT images. e. Quantitative micro-CT images analysis [492]. Abbreviations: SEM: Scanning electron microscope; HMSCs: Human mesenchymal stem cells; Micro-CT: Micro-computed tomography.

Secondly, nanosurface topography has also been shown to promote implant osseointegration in animal models of critical bone defects, including nanoscale topographies with vertically aligned nano-features and nanotubes. Shirazi et al. [469] developed a nanoscale surface with vertically aligned nano-features (D) having diameters of less than 100 nm, created through oxidation and oxalic acid treatment. This surface topography enhanced the osteogenic differentiation of human MSCs (hMSCs) by upregulating the expression of osteogenesis-related genes, including RUNX2, OSX, and BMP-2. Concurrently, the D surface reduced the expression of inflammatory genes such as TNF-α, IL-1β, and iNOS in BMMs and upregulated the expression of osteogenic-inducible genes such as BMP-2 and OSM, promoting the shift of macrophages toward anti-inflammatory and osteogenic phenotypes. In a critical bone defect model, the D surface demonstrated a higher BIC rate than the OS surface after 21 days, suggesting an advantage in early-stage osseointegration. Ultimately, this nanoscale topography significantly improved osseointegration by promoting hMSCs osteogenesis and modulating the immune response of BMMs (Fig. 6A). Rodrigues et al. [490] fabricated hierarchical surface features by combining sandblasting and acid etching treatments on titanium implants, resulting in discrete nanostructures of 20–30 nm superimposed on a micrometer-scale roughness. The roughness parameter (Ra) of the modified surface was 384 ± 43.1 nm, in contrast to 36 ± 5.7 nm for smooth control surfaces. This nanotopography promoted osteoblast differentiation and bone matrix deposition via the upregulation of the Osterix gene, while simultaneously attenuating inflammation and fostering an immunomodulatory microenvironment conducive to osseointegration. In a mouse femoral critical-sized defect model, these combined effects led to improved osseointegration, evidenced by increased bone volume and higher integration rates. Similarly, Salou et al. [499] employed electrochemical anodization to generate well-organized arrays of titanium oxide nanotubes on titanium alloy implant surfaces, with diameters of 37 ± 11 nm and a thickness of 160 nm. In a femoral condyle defect model in New Zealand White rabbits, these nanostructured surfaces exhibited superior bone-to-implant contact (BIC) and enhanced new bone formation. Pull-out testing and histological evaluation revealed significantly greater mechanical anchorage and bone growth in implants with nanotopography compared to conventionally machined and standard sandblasted acid-etched surfaces. After a 4-week healing period, the nanostructured implants demonstrated markedly improved BIC and mechanical fixation.

In addition, a combination of micron- and nanoscale surface topography can also promote the osseointegration of implants in animal models of critical bone defects. Hyzy et al. [491] fabricated Ti-6Al-4V alloy implants using direct metal laser sintering and subjected the surfaces to sandblasting and acid etching treatments. This process created surface topographies with both micro- and nanoscale roughness, along with a high degree of hydrophilicity, which promoted the secretion of local factors, such as osteocalcin, OPG, VEGF, FGF2, and BMP-2, by enhancing the proliferation and differentiation of osteoblasts. As a result, the BIC area significantly increased in a New Zealand White rabbit femur model, ultimately facilitating osseointegration in a critical bone defect model. Similarly, Palmquist et al. [500] used a laser partial modification method to build a micro- and nanoscale surface structure on titanium implants, specifically by treating the threaded valley region of the implant. This surface topology, which combined both micron and nano features, enhanced osseointegration by promoting osteoblast adhesion and differentiation. In a model of extensive bone defects at the femoral and tibial sites of New Zealand White rabbits, the laser-modified implants demonstrated higher removal torque values than conventional machined implants after a 6-month healing period, indicating superior osseointegration.

Furthermore, natural bionic surface topographies, such as those inspired by fish scales, have also been shown to promote implant osseointegration in critical bone defect models. Qin et al. [492] extracted fish scales with natural ridged micropatterns through decellularization and de-collagenization treatments, preserving the characteristic ridged patterns and mineralized collagen components on the surface. These surface features guided cell alignment and migration, while promoting osteoblast adhesion, proliferation, and differentiation. Specifically, the ridged micropatterns on the fish scale surface induced osteogenic differentiation of BMSCs through the Wnt/β-catenin signaling pathway, increasing the expression of osteogenesis-related proteins such as RUNX2, ALP, osteocalcin, and type I collagen. Additionally, the micropatterned and mineralized collagen components on the surface of fish scales facilitated macrophage polarization towards the M2 phenotype, promoting the secretion of anti-inflammatory cytokines (e.g., IL-4 and IL-10) and improving the bone immune microenvironment. In a rat femoral defect model, implantation of fish scales significantly promoted new bone formation and osseointegration. Micro-CT and histological analyses revealed that the fish scale group had superior outcomes compared to the control group in terms of new bone formation at 3 and 6 weeks, with results comparable to the Bio-Oss® bone material commonly used in clinical practice (Fig. 6B). Xiao et al. [493] utilized grass carp scales as raw material, modifying them through decellularization and epigallocatechin gallate treatment. The modified fish scales were combined with mineralization treatment to create a 5R-E-DCFS membrane with a functional gradient bone-like structure. This membrane mimicked the outer and inner layers of natural bone, with decreasing mineralization from the outside to the inside. The 5R-E-DCFS membrane promoted the adhesion, proliferation, and osteogenic differentiation of BMSCs and upregulated the expression of osteogenesis-related genes and proteins, such as RUNX2, COL-1, OCN, and OPN. In a rat model of extensive cranial bone defects, the 5R-E-DCFS membrane significantly enhanced bone defect repair, as evidenced by higher bone volume/tissue volume ratios, increased bone mineral density, and more mature neoplastic bone tissue. HA bioscaffolds with micro- and nanocomposite structures, which mimic the microstructure of natural bone tissues, have also been shown to provide optimal growth conditions for cells, closely resembling the physiological environment. This significantly promotes the healing of bone defects [501,502]. While clinical studies on critical bone defects remain limited, the successful outcomes of animal experiments provide an important theoretical foundation for future clinical applications.

### 5.2 Dental implants

**Fig. 7:**
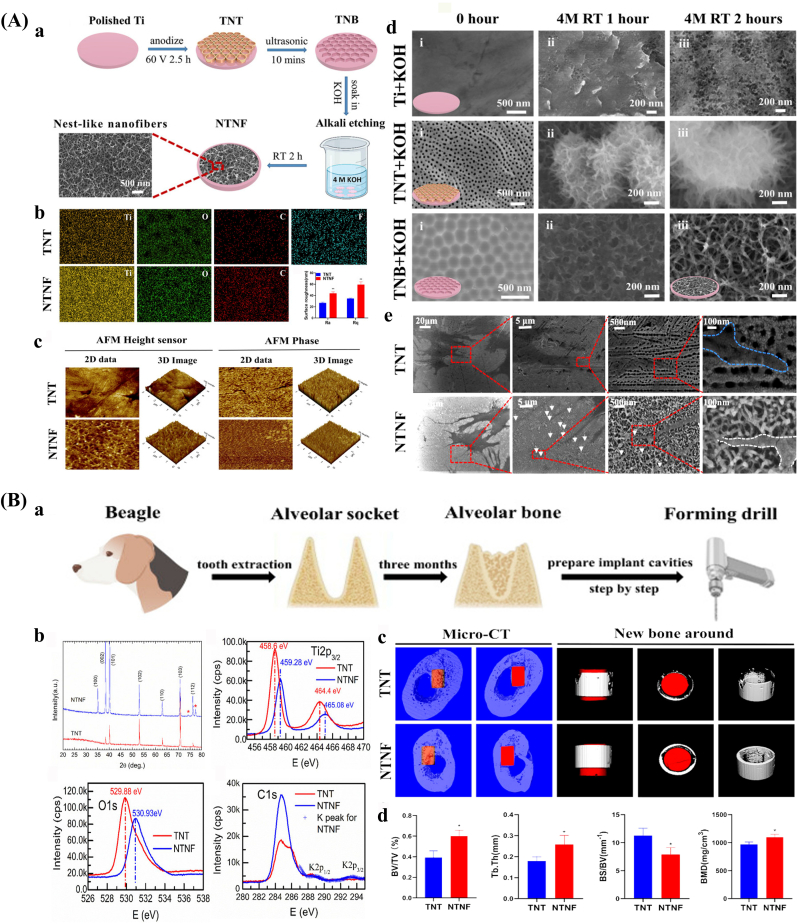
Topology-based implants for in vivo treatment of dental implants. (A) Nested nanofiber structure of titanium nano bowls. a. Preparation schematic. b. EDS mapping images and surface roughness. c. 2D and 3D AFM images. d. FE-SEM images e. SEM image of BMSCs at 24 h [494]. (B) Nanofiber structure promotes osteointegration of Beagle dog Implants. a. Schematic illustration. b. XRD patterns and XPS patterns. c. Micro CT images. d. BV/TV, Tb.Th, BS/BV and BMD [494]. Abbreviations: TNT: TiO_2_ nanotubes; TNB: TiO_2_ nanobowl; NTNF: Nest-like titanite nanofiber structure; AFM: Atomic force microscope; EDS: Energy dispersive spectroscopy; FE-SEM: Field-emission scanning electron microscope; XRD: X-ray diffraction; XPS: X-ray photoelectron spectroscopy; BV/TV: Bone volume/total volume; Tb.Th: Trabecular thickness; BS/BV: Bone surface/bone volume; BMD: Bone mineral density.

The importance of surface morphology for osseointegration has been demonstrated in both animal models of jaw defects and clinical trials involving dental implants. In animal model studies, Ahmed et al. [503] created a medium rough surface (Sa = 1.49 μm) on titanium implants using TiO_2_ sandblasting and acid etching, along with a micro-rough surface (Sa = 0.36 μm) created by acid etching alone. Compared to the medium rough surface, the micro-rough surface more significantly promoted cell adhesion and proliferation, thereby enhancing osteoblast activity more effectively. Additionally, the micro-rough surface showed superior osseointegration in both radiological and histological evaluations, ultimately improving implant osseointegration in a Labrador model of extensive mandibular bone defects. Sun et al. [494] developed a nested titanate nanofiber structure on the surface of commercially pure titanium dental implants using a combination of electrochemical anodization and alkaline etching. In vitro studies demonstrated that this nanostructured surface significantly enhanced the adhesion, proliferation, and viability of BMSCs, and effectively promoted their differentiation into osteoblasts, as evidenced by the upregulation of key osteogenic markers, including ALP, RUNX2, OSX, COL1, and OCN. In vivo evaluation using an alveolar bone implantation model in Beagle dogs revealed that implants featuring the nested nanofiber architecture exhibited significantly improved bonding with surrounding bone tissue compared to conventional implants. The enhanced osseointegration observed suggests strong potential for clinical application (Fig. 7).

In clinical trials, the surface morphology of dental implants plays a crucial role in bone healing. Specifically, the hydrophilicity and hydrophobicity of implant surfaces are closely associated with osseointegration, with hydrophilic surfaces generally being more favorable for osseointegration. Niklaus et al. [60] surgically implanted chemically modified moderately rough hydrophilic (SLActive) and moderately rough hydrophobic (SLA) implants into the posterior molar region of 49 volunteers and assessed the rate and extent of osseointegration at 7, 14, 28, and 42 days. The results showed that BIC was significantly higher around the SLActive implants compared to the SLA implants at both 2 and 4 weeks (14.8 % vs. 12.2 % and 48.3 % vs. 32.4 %, respectively), indicating superior osseointegration on the SLActive surfaces. Additionally, the roughness of the implant surface topography is another important factor influencing osseointegration outcomes. Daniel et al. [58] conducted a 10-year retrospective clinical study to evaluate the survival and success rates of 511 dental implants with micron and submicron roughness and demonstrated that none of the 511 implants fractured over the 10-year period. However, six implants (1.2 %) were lost, two implants (0.4 %) showed signs of sepsis at the 10-year examination, and seven implants (1.4 %) had a history of peri-implantitis during the 10-year study period. Despite this, these implants exhibited healthy peri-implant soft tissue at the final examination.

Dental implant surface morphology has also been linked to bone resorption following periodontitis treatment [504]. Alberto et al. [505] conducted a retrospective evaluation to examine inter-occlusal bone resorption (CBL) after the implantation of exo-hexagonal implants (EHI) with different surface micromorphologies in the mandibular posterior region of patients with a history of peri-implantitis, who received periodontal support. The study, with a follow-up period of 1–6 years, revealed that anodized implants performed better than turned and acid-etched surfaces in reducing inter-occlusal bone resorption. Overall, surface morphology is an important factor for the osseointegration of dental implants. These findings provide an important theoretical basis and clinical reference for the effective application of dental implants in clinical practice.

### 5.3 Diabetic bone defects

**Fig. 8:**
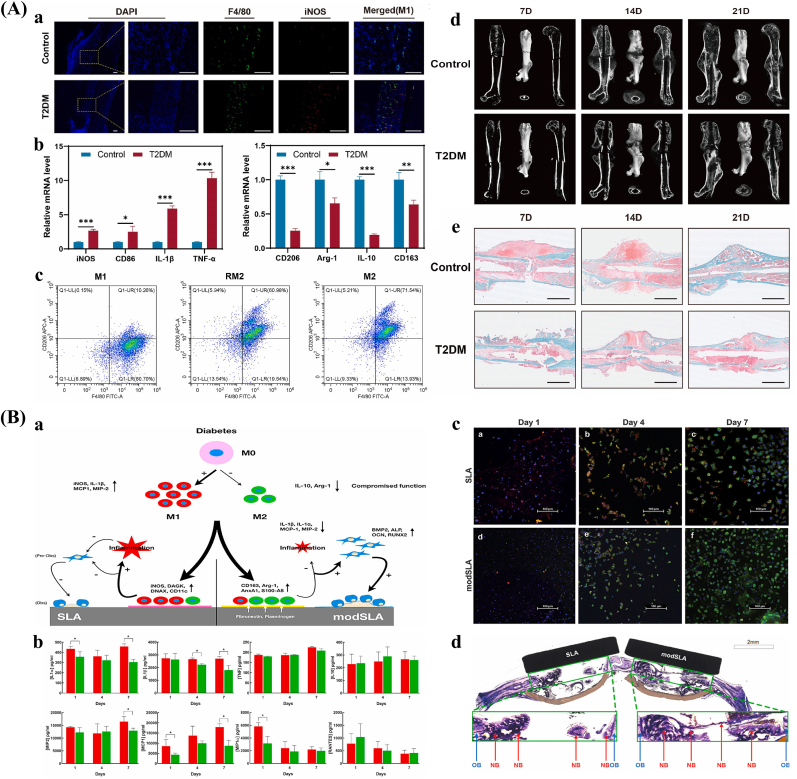
Topology-based implants for in vivo treatment of diabetic bone defects. (A) M2 macrophage-derived exosomes promote diabetic fracture healing by acting as an immunomodulator. a. Localization of M1 macrophages. b. Macrophage mRNA expression levels. c. FCM analysis. d. Micro-CT images. e. SOFG staining of healing tissue sections [507]. (B) Re-establishment of macrophage homeostasis by titanium surface modification in type II diabetes promotes osseous healing. a. Diagram of the molecular mechanism. b. Cytokine secretion profile of M1-polarised GK macrophages. c. Immunofluorescence images. d. Toluidine blue-stained histologic sections [65]. Abbreviations: M0: Unstimulated macrophage; Pre-Obs: Pre-osteoblasts; Obs: Osteoblasts; GK: Goto kakizaki; SLA: Sandblast; modSLA: Modified hydrophilic sandblast; FCM: Flow cytometry; Micro-CT: Micro-computed tomography; SOFG: Standards and ontologies for functional genomics.

Animal studies have demonstrated that implant surface morphology plays a critical role in the repair of bone defects under diabetic conditions. Zhou et al. [66] evaluated the performance of implants with micro-rough surfaces (SLA surfaces), produced by sandblasting and acidetching, exhibiting an average surface roughness of approximately 2.5 μm. These microstructured surfaces were shown to enhance osseointegration by promoting both osteoclast and osteoblast adhesion and proliferation. The increased roughness facilitated cellular interactions, thereby supporting greater new bone formation. In a type 2 diabetic rat tibial defect model, SLA-modified implants exhibited significantly improved osseointegration, as reflected by increased bone-to-implant contact (BIC) and greater newly formed bone volume. In the context of type 2 diabetes (T2D), macrophage function is impaired, particularly with reduced polarization toward the M2 phenotype, which is associated with anti-inflammatory and tissue-repair functions in bone healing [506,507] (Fig. 8A). To address this issue, Ryan S.B. Lee et al. [65] developed hydrophilic nanostructured surfaces (modSLA) through sandblasting and acid-etching techniques. Their study demonstrated that the modSLA surface promoted macrophage polarization toward the M2 phenotype, increased IL-10 secretion, and reduced pro-inflammatory cytokine production under diabetic conditions. Furthermore, modSLA surfaces activated macrophage autophagy by inhibiting the PI3K/Akt/mTOR signaling pathway, thereby further facilitating M2-type polarization. As a result, implants with modSLA surfaces significantly improved BIC and enhanced new bone formation and healing in diabetic rat models (Fig. 8B).

Although clinical studies on the influence of implant surface morphology in diabetic bone defect repair remain limited, findings from preclinical models offer a strong theoretical foundation for future clinical applications. Optimization of implant surface morphology and bioactivity may lead to the development of more effective bone repair materials to address the high prevalence of bone healing complications in patients with diabetes.

### 5.4 Osteoporotic bone defects

**Fig. 9:**
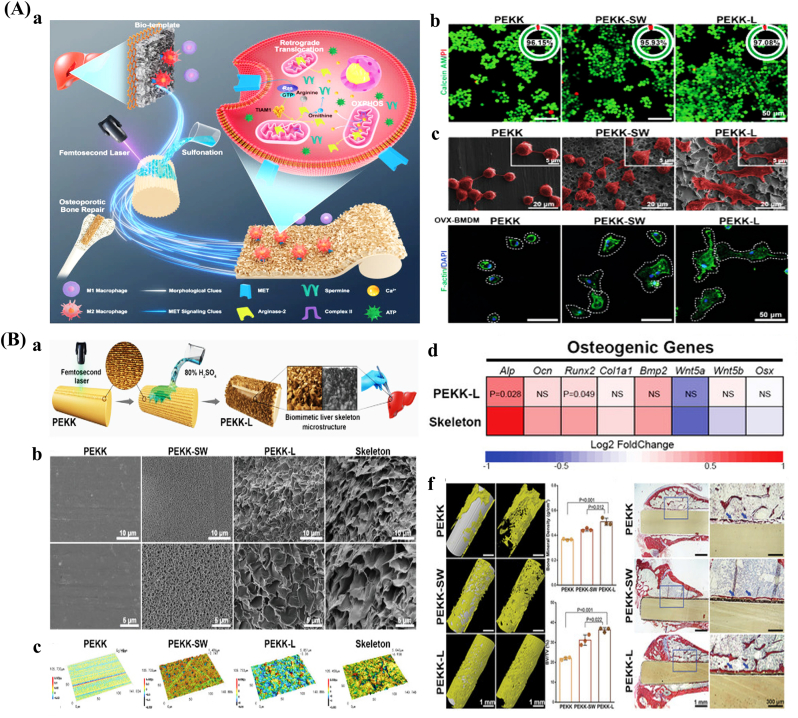
Topology-based implants for in vivo treatment of osteoporotic bone defects. (A) Liver-inspired polyetherketoneketone scaffolds. a. Schematic illustration. b. Live/dead fluorescence, SEM images and cytoskeleton of RAW264.7 cells [495]. (B) Polyetherketoneketone Scaffolds Promote Osteoporotic Osteointegration. a. Schematic illustration. b. SEM images. c. 3D surface optical profiles. d. Expression of osteogenic genes in BMSCs. f. 3D images of micro-CT, quantitative analysis of bone density and bone volume fraction, and Van Gieson staining [495]. Abbreviations: SEM: Scanning electron microscope; PEKK: Polyetherketoneketone; PEKK‐L: Sulfonating PEKK with 80 % H_2_SO_4_; PEKK‐SW: Sulfonating PEKK with 98 % H_2_SO_4_.

Animal experiments have demonstrated that implant surface morphology plays a critical role in promoting bone regeneration in osteoporotic bone defects, even in the absence of pharmacological intervention. Ethan et al. [67] developed microrough SLA surfaces through sandblasting and acid-etching and further modified these surfaces to generate nanostructured SLAnano and hydrophilic mSLA variants. These modified surfaces significantly enhanced osteoblast activity by activating the PI3K/Akt and Wnt signaling pathways, leading to the upregulation of osteogenic markers such as OCN, OPN, and BMP-2. Concurrently, they inhibited osteoclast activity by increasing the expression of osteoprotegerin (OPG). In a femoral implantation model using ovariectomized osteoporotic rats, implants with these surface modifications demonstrated substantial improvements in osseointegration, as evidenced by increased BIC and higher removal torque values (RTVs). In a related study, Gu et al. [495] engineered a three-dimensional petal-like surface (PEKK-L) on polyetherketoneketone (PEKK) scaffolds using femtosecond laser etching followed by sulfonation. This surface mimicked the extracellular matrix structure of hepatic tissue and featured microgrooves, islands, and micropores with an average roughness of approximately 0.884 μm. The PEKK-L surface activated the MET and Ras/TIAM1 signaling pathways, thereby promoting macrophage polarization toward the M2 phenotype, enhancing anti-inflammatory cytokine secretion, and reducing pro-inflammatory cytokine expression. Moreover, the PEKK-L surface improved macrophage immunosensitivity, increasing responsiveness to low-concentration stimuli. In an osteoporotic rat femoral defect model, PEKK-L scaffolds significantly enhanced new bone formation, increased BIC, and improved histological bone parameters, thereby promoting osseointegration under osteoporotic conditions (Fig. 9).

Moreover, porous implant morphologies have demonstrated promising outcomes in osteoporotic models. For instance, Zhu et al. [496] fabricated biomimetic porous magnesium alloy scaffolds with 75 % porosity using hot-pressing and sintering techniques. These scaffolds were designed with a cylindrical geometry (3 mm in diameter and height) and contained randomly distributed pores of varying diameters, resulting in a highly porous and sparse structure. This architecture provided mechanical support while offering sufficient space to facilitate new bone ingrowth. In vitro, the scaffold promoted osteoblast proliferation and enhanced the migration, proliferation, and osteogenic differentiation of BMSCs. Mechanistically, the scaffold activated the Wnt/β-catenin signaling pathway, specifically upregulating the expression of Wnt5a, β-catenin, and BMP-2, thereby promoting bone regeneration at the defect site. In osteoporotic rat femoral defect models, the bionic scaffold significantly enhanced bone repair, as indicated by increased trabecular number, trabecular thickness, and trabecular connectivity density. These outcomes collectively supported improved implant osseointegration under osteoporotic conditions and provide a valuable theoretical basis and clinical reference for advancing the treatment of osteoporotic bone defects through the optimization of implant surface topography.

## 6 Challenges and outlook

**Fig. 10:**
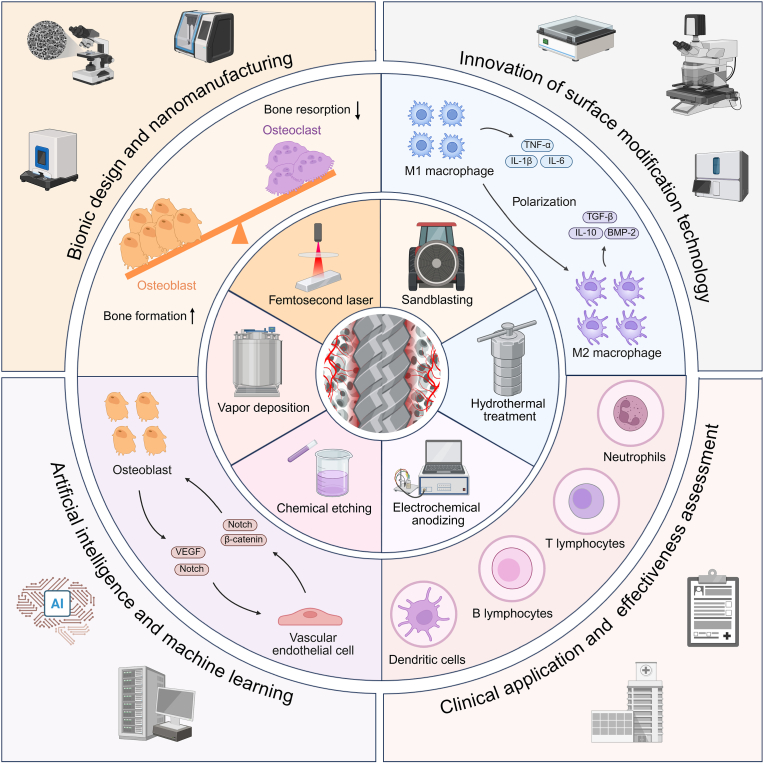
Future prospects for topography-based bone implants. Created in BioRender. Abbreviations: BMP-2: Bone morphogenetic protein 2; VEGF: Vascular endothelial growth factor; TNF-α: Tumor necrosis factor alpha; IL-1β: Interleukin 1 beta; TGF-β: Transforming growth factor beta; IL-10: Interleukin 10.

Although surface morphological modification of bone regeneration implants has demonstrated substantial potential in preclinical research, several challenges remain in translating these findings to clinical practice. Future exploration in this field could focus on the interdisciplinary integration of material science, biology, and engineering, as well as the application of innovative technological tools to optimize implant design and functionality (Fig. 10).

### 6.1 Challenges

Although surface topography holds significant potential in bone regeneration implants, several challenges remain in its practical application. One key issue is achieving the optimal balance between different surface morphologies, such as nanostructures, micron structures, and porous structures [29,469,508,509]. While nanostructures can substantially promote osteoblast adhesion and differentiation, excessive nanoroughness may lead to increased apoptosis [510,511]. Furthermore, while porous structures increase surface area and enhance cell adhesion, the optimal pore size and porosity still require further investigation to ensure effective functionality [[512], [513], [514]]. Therefore, finding a way to promote osseointegration while avoiding excessive apoptosis and inflammatory responses is a pressing concern in current research.

In translating laboratory findings to clinical applications, implant surface morphology must be designed to meet diverse clinical needs. There are variations in how different patients' bone conditions (e.g., osteoporosis, diabetic bone defects) respond to implant surface topography [[515], [516], [517], [518]]. For instance, in patients with osteoporosis, surface topography needs to activate the Wnt/β-catenin signaling pathway more efficiently to promote osteogenesis [519,520]. Additionally, the increasing demand for personalized medicine requires that implant surface morphology be tailored to individual patient characteristics [[521], [522], [523]]. However, current technologies face significant challenges in achieving this level of personalization, such as high manufacturing costs and long production cycles [521,[524], [525], [526]]. Therefore, maintaining a balance between mass production and personalization remains an important direction for future research [527].

### 6.2 Outlook

In the future, research on the surface morphology of bone regeneration implants will increasingly focus on the integration of multiple disciplines and the application of innovative technologies [524,525]. Cross-collaboration between materials science, biology, and medicine could provide new perspectives for surface topography design. For example, advanced nanofabrication techniques can be used to develop more complex surface structures. Additionally, artificial intelligence (AI) and machine learning can assist in optimizing the selection and preparation of surface topographies by building predictive models, improving research and development efficiency, and providing a better understanding of material property changes [495,[528], [529], [530], [531]]. In clinical applications, future studies could emphasize the long-term effects of implant surface morphology. With the continuous advancement of technology, the service life and biocompatibility of implants may be further enhanced [532,533]. Moreover, clinical studies should increasingly examine implant performance in various pathological conditions, such as diabetes, osteoporosis, and infections, within complex environments [534]. A standardized clinical evaluation system, once established, will enable a more comprehensive assessment of implant performance, thereby providing clinicians with a more reliable foundation for treatment decisions [535]. This approach could lead to the development of bone regeneration implants that address the diverse needs of clinical practice and offer enhanced treatment options for patients.

#### 6.2.1 Bionic design and nanomanufacturing

Bionic design and nanofabrication techniques enable the development of surface morphologies with more complex structures that closely mimic the microenvironment of natural bone tissue [525,536]. Long-term clinical studies have demonstrated that these biomimetic surface morphologies can significantly enhance cellular infiltration and proliferation, thereby promoting bone regeneration and improving implant stability [537]. For instance, 3D printing technology allows the fabrication of hierarchical porous scaffolds with nanofibrous structures, which not only significantly enhance cellular infiltration and proliferation rates but also effectively guide osteogenic differentiation and ECM deposition [[538], [539], [540]]. Future research could further optimize these biomimetic surface morphologies by precisely controlling the size and distribution of nanostructures to enhance the biocompatibility and osseointegration of implants, which may help develop more efficient bone regeneration implants with longer lifespans.

#### 6.2.2 Innovation of surface modification technology

In the field of surface modification technology, the combination of laser, electron beam, and ion beam techniques offers an effective strategy for constructing implant surface morphologies, representing a promising direction for future modifications [28,541]. Among these, laser processing technology stands out as a non-contact method with several advantages, including the absence of impurities, high precision, excellent repeatability, high penetration strength, and low cost [542,543]. By adjusting parameters such as laser fluence, pulse count, and wavelength, it is possible to precisely control the dimensions of grooves and induce various periodic microgroove and nanopore cluster-like structures on bone implant surfaces, thereby creating biomimetic surface morphologies [[544], [545], [546], [547]]. These innovative surface modification techniques play a crucial role in advancing the development of high-performance bone regeneration implants.

#### 6.2.3 Application of AI and machine learning

The optimization of material surface morphology selection and preparation, along with the development of accurate predictive models through AI and machine learning techniques, can enhance research and development efficiency, reduce costs, and provide a better understanding of the relationship between surface morphology and biological response [[529], [530], [531]]. For example, machine learning can be applied to predict the effects of different surface topographies on cellular behavior, guiding surface design decisions [72,495]. Additionally, Generative adversarial networks (GANs), a class of deep learning models, can generate novel surface topography designs by training competing models to create surface topographies with specific properties, ultimately improving the biocompatibility and osseointegration of implants [[548], [549], [550]].

#### 6.2.4 Clinical application and long-term effectiveness assessment

Assessing the long-term effects of implant surface morphology is critical to enhancing implant longevity and biocompatibility. Long-term clinical studies would provide valuable insights into how surface morphology impacts bone regeneration and implant stability [532,533]. Future studies may focus more on the behavior of implants with various surface morphologies in different pathological conditions, such as diabetes, osteoporosis, and infections, to develop more targeted implants [534,551], as understanding implant performance in these complex pathological conditions would allow for the further optimization of implant designs, improving their effectiveness and safety across different clinical settings [552]. Lastly, establishing a standardized clinical evaluation system could enable a comprehensive assessment of implant performance, providing clinicians with a reliable reference and facilitating comparisons and communication between different studies [535,553].

## CRediT authorship contribution statement

**Weiwei Guo:** Writing – review & editing, Writing – original draft, Visualization, Supervision, Investigation. **Zuge Yang:** Writing – review & editing, Writing – original draft, Visualization, Supervision, Investigation. **Fuwei Liu:** Writing – review & editing, Writing – original draft, Supervision. **Jianye Song:** Writing – original draft, Investigation. **Wenhao Yang:** Writing – original draft, Investigation. **Yunpeng Li:** Writing – review & editing, Funding acquisition, Supervision, Resources. **Wenhui Hu:** Writing – review & editing, Supervision, Resources, Conceptualization. **Kun Wang:** Writing – review & editing, Supervision, Project administration, Funding acquisition, Conceptualization.

## Declaration of competing interest

The authors declare that they have no known competing financial interests or personal relationships that could have appeared to influence the work reported in this paper.

## Data Availability

No data was used for the research described in the article.

## **Abbreviations:**

**BMSCs**: Bone marrow mesenchymal stem cells
**VEGF**: Vascular endothelial growth factor
**lncRNAs**: Long non-coding RNAs
**TNF**: Tumor necrosis factor
**NF-κB**: Nuclear factor-κB
**IL-1β**: Interleukin 1β
**TGF-β**: Transforming growth factor-β
**BMP-2**: Bone morphogenetic protein-2
**DCs**: Dendritic cells
**BMMs**: Bone marrow-derived macrophages
**ECM**: Extracellular matrix
**MSCs**: Mesenchymal stem cells
**RANKL**: Receptor activator of nuclear factor-κB ligand
**OPG**: Osteoprotegerin
**TLRs**: Toll-like receptors
**BIC**: Bone-implant contact
**SLM**: Selective laser melting
**EBM**: Electron beam melting
**CVD**: Chemical vapor deposition
**FAK**: Focal adhesion kinase
**RGD**: Arginine-glycine-aspartate
**ALP**: Alkaline phosphatase
**BDNF**: Brain-derived neurotrophic factor
**NGF**: Nerve growth factor
**DRG**: Dorsal root ganglion
**HA**: Hydroxyapatite
**ERM**: Ezrin/Radixin/Moesin
**NETs**: Neutrophil extracellular traps
**SDF-1**: Stromal Cell-derived Factor 1
**cPLA2**: Cytosolic phospholipase A2
**PL**: Polishing
**DAE**: Double-acid-etching
**DCD**: Deposition of nanocrystals
**hMSCs**: Human MSCs
**EHI**: Exo-hexagonal implants
**T2D**: Type 2 diabetes
**RTVs**: Removal torque values
**PEKK**: Polyetherketoneketone
**AI**: Artificial intelligence
**GANs**: Generative adversarial networks.
